# Defect‐Guided Assembly of Aperiodic and Flexible Metal–Organic Frameworks From Pre‐Formed Cages

**DOI:** 10.1002/anie.9270871

**Published:** 2026-06-26

**Authors:** Francisco Sánchez‐Férez, Akim Khobotov‐Bakishev, Borja Ortín‐Rubio, Cornelia von Baeckman, Laura Hernández‐López, Alba Cortés‐Martínez, Robert A. I. Paraoan, Roberto Boada, Judith Juanhuix, Felipe Gándara, Andrew L. Goodwin, Arnau Carné‐Sánchez, Daniel Maspoch

**Affiliations:** ^1^ Catalan Institute of Nanoscience and Nanotechnology (ICN2) CSIC and The Barcelona Institute of Science and Technology Campus UAB Barcelona Spain; ^2^ Department of Chemistry Faculty of Science Universitat Autònoma de Barcelona (UAB) Barcelona Spain; ^3^ Inorganic Chemistry Laboratory Department of Chemistry University of Oxford Oxford UK; ^4^ ALBA Synchrotron Light Facility Barcelona Spain; ^5^ Materials Science Institute of Madrid (ICMM) Consejo Superior de Investigaciones Científicas (CSIC) Madrid Spain; ^6^ ICREA Pg. Lluís Companys 23 Barcelona Spain

**Keywords:** defects engineering, mesoporous, metal–organic frameworks, methanol adsorption

## Abstract

Controlling the spatial distribution of defects in metal–organic frameworks (MOFs) remains a fundamental challenge in defect engineering. Here, we report a cage‐directed assembly strategy that enables the programmed introduction of topologically correlated defects and induces aperiodicity in HKUST‐1‐type frameworks. Pre‐synthesised Rh(II) metal–organic cages or polyhedra (MOPs) act as persistent cavities that template the formation of HKUST‐1‐based networks containing linker‐induced Cu‐vacancy domains confined within discrete cuboctahedral cavities. The use of dicarboxylate linkers, in which one carboxylate group is missing compared to the original 1,3,5‐benzenetricarboxylate linker, gives rise to nine distinct local defect configurations that are independently distributed throughout the lattice. This results in an aperiodic framework with preserved long‐range crystallinity. The resulting materials exhibit hierarchical micro–mesoporosity, reversible loss and recovery of crystallinity, and a pronounced solvent‐induced breathing response. This work demonstrates that combining pre‐formed cages with linkers with reduced connectivity can be a new strategy to localise defects and access aperiodic MOFs with emergent structural adaptability.

## Graphical abstract

A cage‐directed strategy is used to program topologically correlated defects and induce aperiodicity in HKUST‐1‐type MOFs. A missing carboxylate group in the linker drives the formation of Cu‐vacancy domains, while Rh(II) cages spatially confine these defects. The resulting MOFs are crystalline yet aperiodic, with hierarchical porosity, reversible crystallinity, and solvent‐driven breathing.

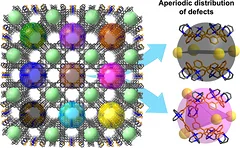

## 1 Introduction

Defect engineering in metal–organic frameworks (MOFs) has garnered escalating interest in recent years [1, 2, 3]. Defects within MOFs are defined as sites that disrupt the regular periodic arrangement of atoms or ions within the crystalline framework, either due to missing elements or their displacement. These defects manifest on different geometric scales, encompassing point, line, and planar configurations [4]. Among these, point defects have emerged as a focal point of extensive research across diverse disciplines owing to their profound influence on MOF properties [5]. Both experimental and theoretical investigations have revealed that defects can significantly alter the physical and chemical properties of reticular materials, thereby establishing them as a versatile tool for tailoring material characteristics [4]. This capacity leads to the introduction of new functionalities and the enhancement of performance metrics such as adsorption capacity [6, 7, 8], band structures [9, 10], conductivity [11, 12], mechanical responses [13], and catalytic activity [14, 15, 16, 17].

Among the most studied methodologies for introducing defects into MOFs are *de novo* synthesis and post‐synthetic treatments [5]. In *de novo* synthesis, common techniques include the use of modulators [15, 18, 19, 20], or mixed‐ligand synthesis [6, 16, 21, 22], aimed at deliberately incorporating defects during the framework formation process. Conversely, post‐synthetic treatments encompass a range of strategies, including the exchange of metal ions and/or linkers in the material [23, 24], utilisation of etching agents such as acids [25, 26, 27], bases [28, 29], or salts [30], as well as the application of thermal [31, 32, 33] or plasma treatments [34, 35] to induce defects *ex post facto*.

Defects introduced by such methodologies were long believed to be randomly distributed within the crystal lattice of MOFs. However, recent studies have revealed a structurally correlated effect when the connectivity of the linker is reduced with respect to the ideal linker for a given network [36]. The vacancies generated through this approach can influence the likelihood of neighbouring linkers creating correlated defective nanoregions, which can be systematically controlled by the manipulation of relative concentrations between modified and standard linkers [37, 38, 39]. Moreover, a recent mixed ligand synthesis yielded a flexible MOF in which the most probable defect configuration is hypothesised to align one‐dimensionally [40]. Despite these advancements, achieving precise control over the spatial distribution of defects within MOF networks remains a significant challenge.

**FIGURE 1:**
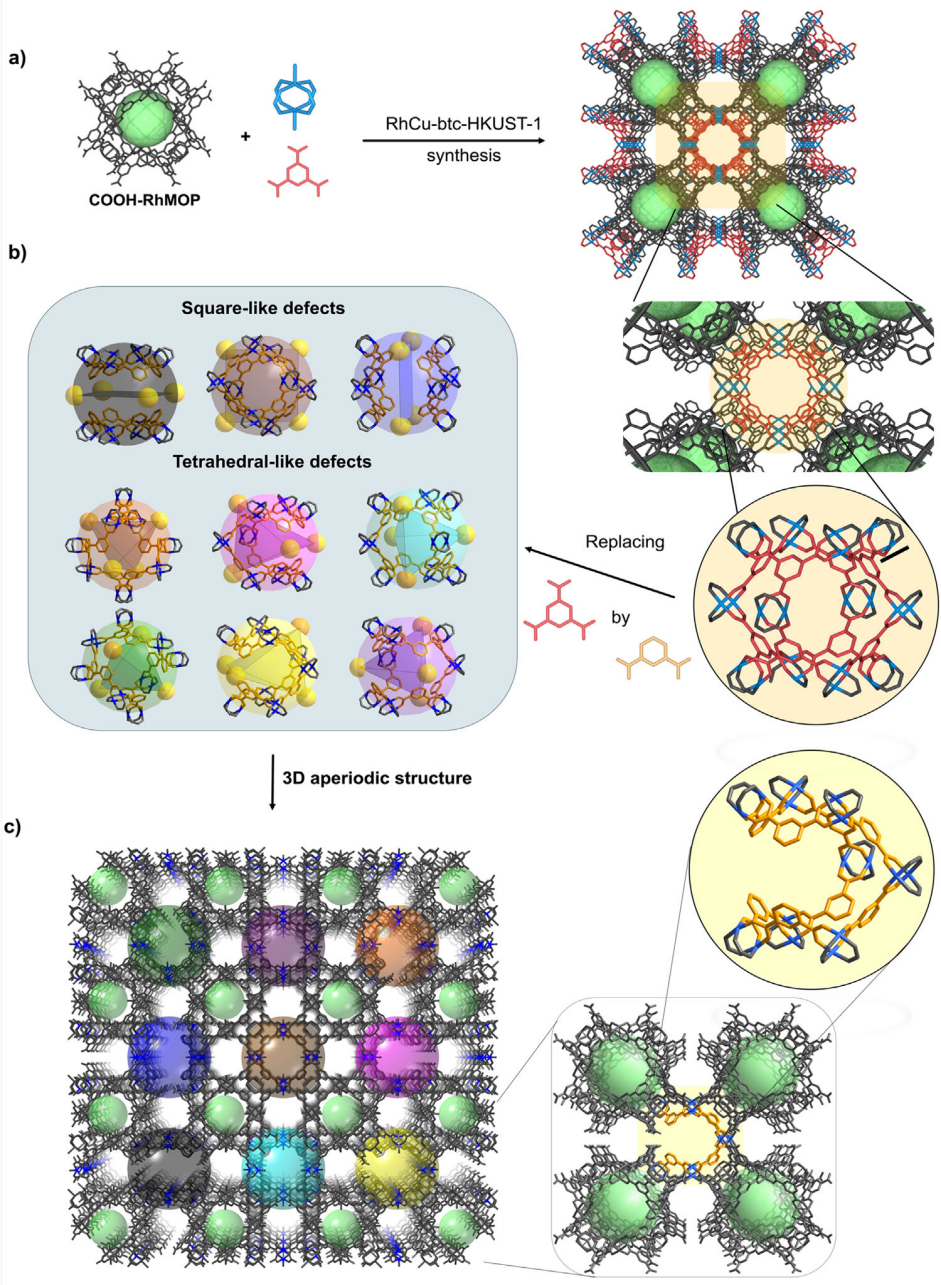
(a) Schematic representation of the synthesis of RhCu‐btc‐HKUST‐1 using the pre‐formed COOH–RhMOP cage, Cu(II), and the 1,3,5‐benzenetricarboxylate (btc) linker. (b) Structural analysis of the expected vacancies generated by replacing btc with 1,3‐benzenedicarboxylate (bdc) in this approach, including the representation of the nine feasible defect configurations within the Cu(II)‐based cuboctahedral cavities (left). (c) Model of the simulated aperiodic HKUST‐1‐type structure (space group Pm–3m), in which the nine distinct local configurations are randomly distributed.

Herein, we propose a new strategy for the precise localisation of topologically related defects within a lattice through the use of structure‐directing agents, leading to the formation of aperiodic frameworks. This approach builds upon our previous work employing metal–organic cages or polyhedra (MOPs) as prefabricated cavities for the synthesis of multi‐component, heterometallic, and heteroleptic MOFs isoreticular to HKUST‐1, named RhCu‐btc‐HKUST‐1 (btc = 1,3,5‐benzenetricarboxylate) [41]. To construct the RhCu‐btc‐HKUST‐1 framework (Figure 1a), we used a pre‐synthesised Rh(II) cuboctahedral MOP [42] functionalised with 24 carboxylic acid groups (hereafter referred to as COOH–RhMOP) [43], together with two additional molecular building blocks (MBBs): the 4‐connected (4‐c) Cu(II) paddlewheel cluster and the 3‐connected (3‐c) btc linker. In this structure, COOH–RhMOP units are quadruple‐bridged by Cu(II) paddlewheel clusters, while btc linkers occupy triangular windows that connect three of these Cu(II)‐based clusters. This connectivity generates a Cu(II)‐based cuboctahedral cavity that interconnects eight COOH–RhMOP cavities arranged in a cubic fashion. Each Cu(II)‐based cavity is defined by 12 Cu(II) paddlewheel clusters connected through 14 btc linkers from the COOH–RhMOP and 8 additional btc linkers incorporated during synthesis. Overall, within this HKUST‐like architecture, the propagation of the MOPs relies entirely on the Cu(II) paddlewheel units, whereas the btc linkers primarily provide structural stabilisation and template the formation of these clusters.

Given that btc linkers play a secondary role in the growth of this framework, we sought to understand what would happen if a defect was introduced by eliminating one of the three carboxylate groups in these linkers. Would the structure still form? And if so, would the defect introduced at the linker level propagate throughout the entire framework?

## 2 Results and Discussion

To begin investigating the consequences of this substitution, we first considered the possible structures that could form when using 1,3‐benzenedicarboxylate (bdc) instead of btc (i.e., removing one coordinating group). We hypothesised that, in the newly synthesised framework, all structural components would occupy the same positions as in the original RhCu–btc–HKUST‐1 MOF. This implies that, as described above, the bdc linkers would be located within the Cu(II)‐based cuboctahedral cavities that interconnect eight COOH–RhMOP units arranged in a cubic fashion. Consequently, the defective domain becomes confined within each of these cuboctahedral regions of the lattice, which remain structurally independent from one another (Figure 1b).

Furthermore, the orientation of the bdc linkers within this defective region dictates whether the defects are randomly distributed within the Cu(II)‐based cuboctahedral cavity or adopt a symmetric arrangement. To rationalise this, we performed a structural analysis to identify potential defective architectures that bypass the formation of unstable “T”‐shaped Cu(II) paddlewheel clusters with incomplete coordination, and instead retain only the fully 4‐c paddlewheel nodes as connections between pre‐fabricated cavities [31, 43].

Based on this criterion, our analysis identified nine feasible defect configurations involving the omission of four Cu(II) paddlewheel clusters within the Cu(II)‐based cuboctahedral cavities: three exhibiting a square‐like vacancy arrangement (Figures 1b and S1–S3), and six displaying a tetrahedral‐like vacancy arrangement (Figures 1b and S4–S9). In both families, the orientations of neighbouring bdc linkers are correlated, resulting in well‐defined defect domains within each Cu(II)‐based cuboctahedral cavity. These defects correspond to Cu(II) paddlewheel vacancies that generate internal voids decorated with two uncoordinated carboxylic acids from the COOH–RhMOPs.

Considering these nine configurations, if all Cu(II)‐based cuboctahedral cavities were to adopt the same configuration, a periodic (translational) ordering of defects or vacancies would occur, lowering the symmetry relative to ideal HKUST‐1. In particular, if all Cu(II)‐based cuboctahedral cavities adopted the same square‐like vacancy arrangement, a two‐dimensional framework with space group *P4/mmm* would result, yielding a tetragonal structure (Figure S10). In contrast, if all Cu(II)‐based cuboctahedral cavities adopted the same tetrahedral‐like vacancy arrangement, a periodic three‐dimensional framework with space group *P−4m2* would be formed (Figure S11). However, it is more plausible that all or a subset of these nine defect configurations are independently adopted within individual Cu(II)‐based cuboctahedral cavities, as these cavities are structurally independent from one another. This scenario would lead to an aperiodic structure [44] in which nine distinct local configurations are distributed throughout a three‐dimensional HKUST‐1‐type framework with average space group *Pm−3m* (Figure 1c).

Guided by these theoretical predictions, we next examined whether replacing btc by bdc in the synthesis of the RhCu–HKUST‐1 system would lead to the formation of a crystalline framework, and, if so, which of the predicted structures would be adopted. To this end, we synthesised RhCu–bdc–HKUST‐1, with the formula [COOH–RhMOP(Cu)_16_(bdc)_8_], by heating a mixture of COOH–RhMOP with 16 mol equiv. of Cu(NO_3_)_2_·3H_2_O and 8 mol equiv. of bdc in *N,N*‐dimethylformamide (DMF) at 100°C for 24 h. This solvothermal reaction yielded a colloidal green dispersion. A green crystalline solid (yield: 75%) was isolated by centrifugation, washed with DMF and methanol (MeOH), dried at 85°C for 1 h, and subsequently washed with MeOH and water before being resuspended in MeOH. Synchrotron powder x‐ray diffraction (PXRD) collected on this solid confirmed a pattern nearly coincidental with that of HKUST‐1 or RhCu‐btc‐HKUST‐1 (Figure S12).

Field‐emission scanning electron microscopy (FESEM) analysis of the green solid revealed large particles composed of polycrystalline aggregates of uniform nanocrystallites with an average size of 26 ± 2 nm (Figure S13). Energy‐dispersive x‐ray spectroscopy (EDX) performed on individual nanocrystallites using high‐resolution transmission electron microscopy (HR‐TEM) confirmed the presence of both Rh and Cu within each nanocrystallite (Figure S14). Inductively coupled plasma mass spectrometry (ICP‐MS) measurements on acid‐digested samples revealed a Cu/Rh ratio of 0.69 ± 0.01, in excellent agreement with the expected value for a defective structure missing one‐third of the Cu(II) sites.

**FIGURE 2:**
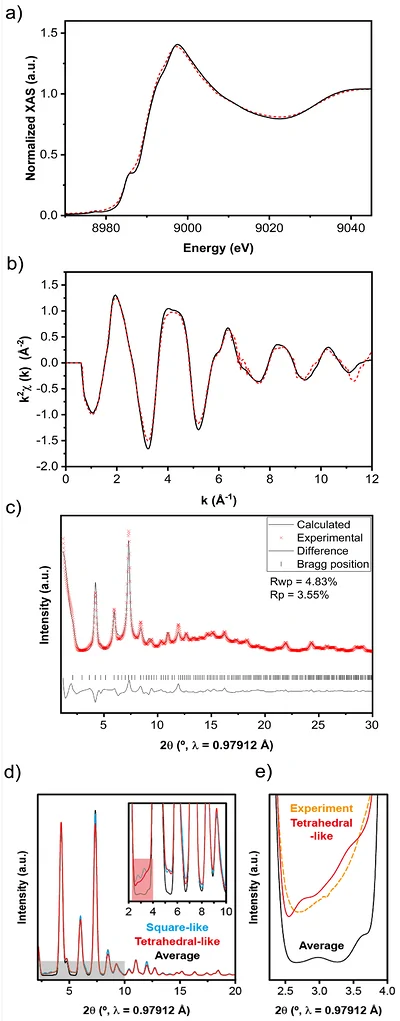
(a,b) Comparative XAS (a) and extended x‐ray absorption fine structure (EXAFS) (b) spectra of RhCu‐bdc‐HKUST‐1 (red dashed line) and HKUST‐1 (black line). (c) Rietveld refinement of RhCu‐bdc‐HKUST‐1. The unit‐cell parameters and atomic position were refined to an aperiodic cubic structure with space group *Pm‐3m*. (d,e) PXRD patterns calculated from atomistic models of RhCu‐bdc‐HKUST‐1 with different paddlewheel vacancy distributions. The black curve shows data for an average‐vacancy model; that in blue a model with randomly‐arranged square‐like defects; that in red a model with randomly‐arranged tetrahedra‐like defects; and that in dash orange the experimental data. Inset of the 2‐10° 2*θ* region highlighting the diffuse scattering observed below the first main peak at 2*θ* ∼ 4.5°.

X‐ray absorption spectroscopy (XAS) confirmed that the coordination environment of Cu(II) in RhCu‐bdc‐HKUST‐1 is equivalent to that in HKUST‐1 and RhCu‐btc‐HKUST‐1, thus corresponding to a non‐defective 4‐c paddlewheel cluster. Furthermore, the presence of undercoordinated Cu(II) paddlewheel clusters (i.e., 3‐c “T”‐shaped paddlewheels) can be excluded based on the analysis of the shoulder feature at the rising absorption edge, which would otherwise appear more intense and shifted to lower energy, as previously reported (Figure 2a,b) [45]. Attenuated total reflectance–Fourier transform infrared (ATR–FTIR) spectroscopy confirmed the presence of free carboxylic acid groups in RhCu–bdc–HKUST‐1. A distinct vibration band at 1705 cm^−^^1^, assigned to the C═O stretching mode, indicates the presence of uncoordinated carboxylic acid functionalities (Figure S15). To further corroborate the MOF composition, the framework was disassembled under basic conditions, and the resulting species were analysed by ^1^H nuclear magnetic resonance (NMR) spectroscopy (Figure S16). These experiments revealed a molar ratio of 8:1 between the bent linker and the prefabricated COOH–RhMOP cavity, in perfect agreement with the composition predicted for our simulated structures. Moreover, these results demonstrate that the COOH–RhMOP unit remains intact during the self‐assembly process, without undergoing ligand exchange or decomposition. In addition, the thermal stability of the solid was evaluated through thermogravimetric analysis (TGA) indicating that the material remains stable up to 340°C (Figure S17).

After confirming the formation of defective RhCu‐bdc‐HKUST‐1, we next sought to determine whether the defect or vacancy sites are periodically or aperiodically distributed throughout the framework by analysing the experimental PXRD data. As discussed above, a periodic (translational) ordering of defects would lower the symmetry with respect to the ideal HKUST‐1 and lead to tetragonal structures in both periodic scenarios: the square (2D) defect arrangement with space group *P4/mmm*, and the three‐dimensional tetrahedral defect arrangement with space group *P*−4*m*2. In contrast, the aperiodic defect arrangement can be captured within the cubic structure of RhCu‐btc‐HKUST‐1 (space group *Pm*−3*m*) by introducing partial occupancies (2/3) for the positions of the metal and carboxylate atoms that form the copper secondary building units. Rietveld refinements were therefore performed with the two periodic models with periodic arrangement of defects as well as with the model with the aperiodic defect distribution (Figures 2c and S18), and a substantially better agreement was obtained for the latter (R*wp* = 6.51%, 5.38%, and 4.83% for the ordered tetrahedral, ordered square, and aperiodic models, respectively). These results suggest that RhCu‐bdc‐HKUST‐1 combines an aperiodic distribution of defects within the Cu‐based cuboctahedral cavities with the translational order imposed by the COOH–RhMOP building blocks (Figure 1c).

Our interpretation is that the paddlewheel vacancies are not randomly arranged but rather correlated within cuboctahedral cavities to give one of the nine defect configurations identified in Figure 1. Such correlations would have a signature in the diffraction pattern in the form of diffuse scattering. By building supercell models of possible defect arrangements, we show that this diffuse component appears as a broad feature centred below the first main Bragg peak at 2*θ* ∼ 4.5°; with a maximum intensity of only about 3% of that of the Bragg peak itself (Figure 2d,e). Our experimental data (Figure 2c) indeed show diffuse scattering in this region that is not accounted for by the Rietveld refinement. These observations are therefore consistent with the defect model proposed, even if our sensitivity to the details of this scattering is not strong. In principle, subtle differences in the diffuse scattering are expected for square‐ and tetrahedral‐like vacancy localizations; however, resolving these experimentally would require high‐quality single‐crystal diffuse scattering measurements (Figures 2d,e and S19). No experimental evidence is observed for correlations between neighbouring defect clusters, which would give rise to additional diffuse‐scattering features beyond those described above.

**FIGURE 3:**
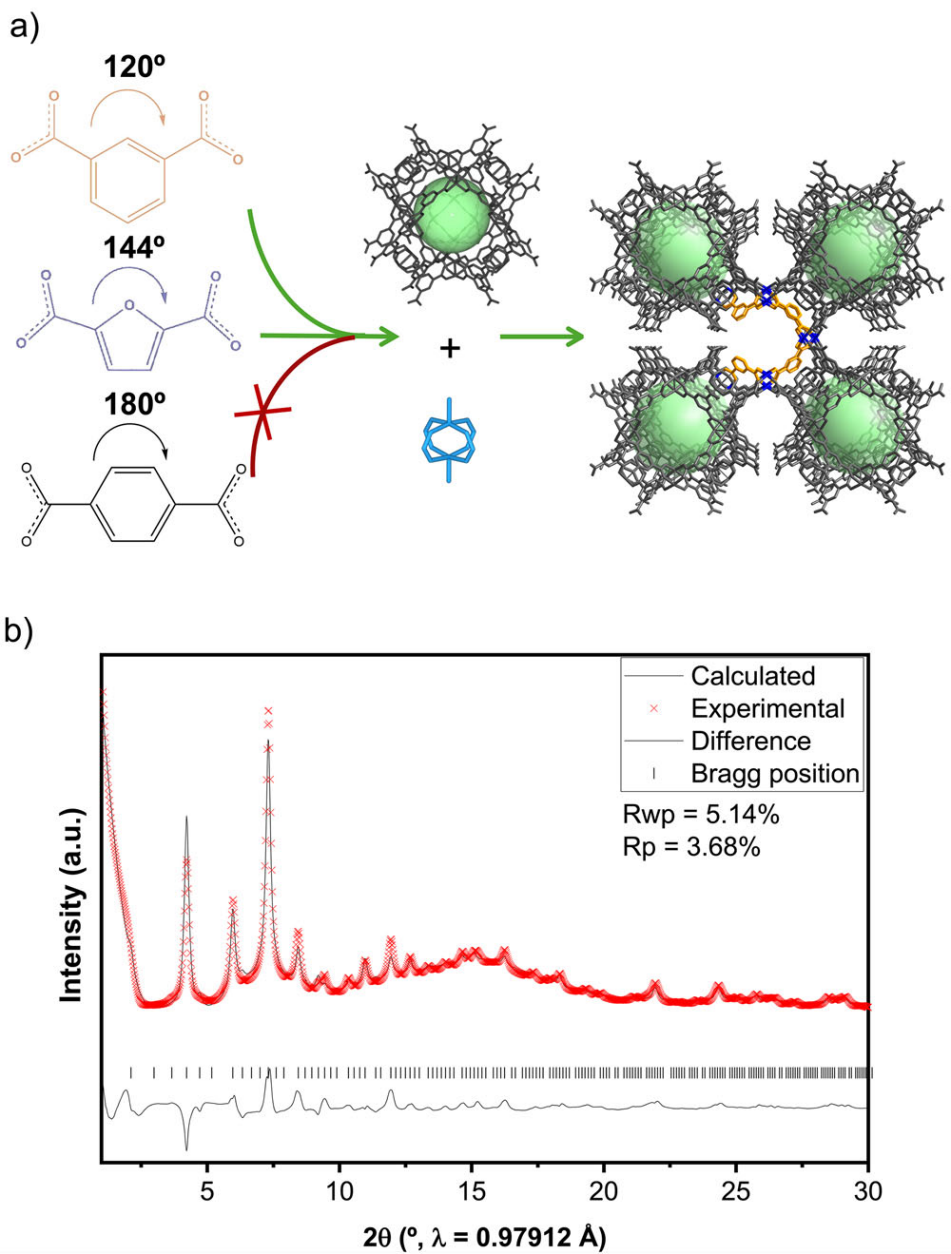
(a) Representation illustrating how the angle of the dicarboxylate linker (bdc, orange; fdc, violet; bdc‐1,4; black) influences the formation of the HKUST‐1‐type structure when combined with COOH–RhMOP and Cu(II). (b) Rietveld refinement of RhCu‐fdc‐HKUST‐1. The unit‐cell parameters and atomic positions were refined using an aperiodic cubic model with space group Pm–3m.

To broaden the scope of our synthetic approach and further assess the influence of the bend angle between the carboxylate groups of the secondary organic linker on the formation of these aperiodic MOFs, we explored the reactivity of two additional linkers with COOH–RhMOP and Cu(II): 1,4‐benzenedicarboxylate (1,4‐bdc), which features a 180° angle between carboxylates, and 2,5‐furandicarboxylate (fdc), which presents a 144° angle, in contrast to the 120° angle in bdc (Figure 3a). In the first case, all attempts to obtain a crystalline material were unsuccessful, consistently yielding amorphous solids. This result indicates that the linear geometry of the 1,4‐bdc linker is incompatible with the relative arrangement of Cu(II) paddlewheels required to sustain the defective RhCu‐bdc‐HKUST‐1 structure. In contrast, the reaction with fdc produced a crystalline framework as in RhCu‐bdc‐HKUST‐1, as confirmed by PXRD, ICP‐MS, FESEM, EDX, XAS, FTIR, ^1^H NMR, and TGA (Figures S20–S25). For this new MOF, Rietveld refinement further confirmed the validity of the aperiodic model (Figure 3b), showing good agreement (R*wp* = 5.14%).

**FIGURE 4:**
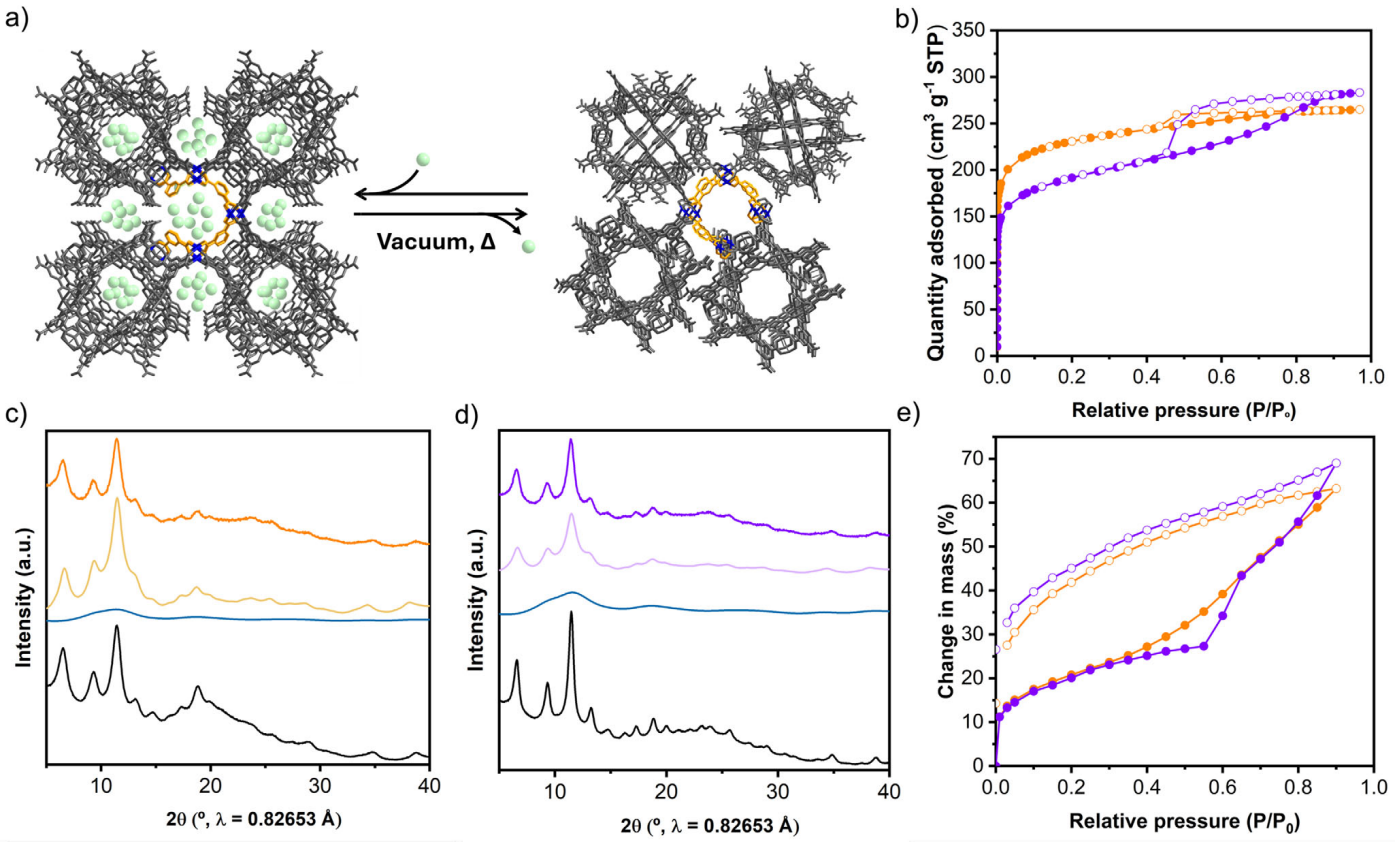
a) Schematic illustration showing the flexible structure of RhCu‐bdc‐HKUST‐1 upon removal or sorption of guest molecules. (b) N_2_ sorption isotherms at 77 K for RhCu‐bdc‐HKUST‐1 (orange) and RhCu‐fdc‐HKUST‐1 (violet). (c,d) PXRD patterns of (c) RhCu‐bdc‐HKUST‐1 and (d) RhCu‐fdc‐HKUST‐1 in the as‐synthesised state (black), after activation (blue), after being immersed in liquid MeOH (light orange in c), and violet in d)) and after exposure to MeOH vapours (orange in c) and violet in d)). (e) MeOH sorption isotherms of RhCu‐bdc‐HKUST‐1 (orange) and RhCu‐fdc‐HKUST‐1 (violet).

The introduction of defects in RhCu‐bdc‐HKUST‐1 and RhCu‐fdc‐HKUST‐1 results in less connected HKUST‐1 frameworks that inherently contain additional potential void volumes. To evaluate the impact of our synthetic strategy on the structural stability and porosity of RhCu‐bdc‐HKUST‐1 and RhCu‐fdc‐HKUST‐1, we first activated the materials using conventional solvent‐exchange followed by thermal vacuum activation, as well as supercritical CO_2_ activation. In both cases, PXRD analysis of the activated solids revealed a complete loss of crystallinity (Figure 4c,d). In contrast to HKUST‐1 or RhCu‐btc‐HKUST‐1, this observation suggests a possible collapse of the framework upon solvent removal (Figure 4a).

To investigate the porosity of the activated phases, N_2_ sorption measurements were carried out at 77 K. Although RhCu‐bdc‐HKUST‐1 and RhCu‐fdc‐HKUST‐1 lose crystallinity upon activation, they remain porous, displaying a type IV isotherm with a sharp uptake at low relative pressures, indicative of microporosity (Figure 4b). The presence of an H2‐type hysteresis loops further evidences mesoporosity, generally associated with pore‐blocking effects, ink‐bottle‐type pores, or disordered and network‐restricted mesoporous domains often arising from structural defects. The Brunauer–Emmett–Teller surface area (S_BET_) was calculated to be 854 m^2^·g^−^^1^ for RhCu‐bdc‐HKUST‐1 and 705 m^2^·g^−^^1^ for RhCu‐fdc‐HKUST‐1 (Figures S26–S29). Pore size distribution (PSD) analysis revealed two main pore populations centred at ∼8 and ∼10‐11 Å, which can be ascribed to the cuboctahedral cavities and to the cavities created between these cuboctahedra (Figure S30). Additional pore populations in the 18–25 Å range confirmed the presence of mesopores.

Remarkably, both RhCu‐bdc‐HKUST‐1 and RhCu‐fdc‐HKUST‐1 exhibited structural flexibility upon adsorption of solvent guest molecules. Indeed, their crystallinity was recovered by immersing the activated materials in various polar solvents such as MeOH, acetone, ethanol, acetonitrile, tetrahydrofuran, and water (Figures S31 and S32). In contrast, immersion in non‐polar solvents such as hexane, toluene, and chloroform did not restore the crystallinity (Figures S33 and S34). To further probe this flexible behaviour, we investigated their MeOH adsorption properties at 298 K. We first confirmed that the activated phases also recover their original crystalline structure upon exposure to MeOH vapours (Figures 4c and 4d). Sorption measurements revealed a clear breathing effect at a relative pressure of ca. 0.55, consistent with the reopening of the framework as crystallinity is recovered. Notably, this breathing behaviour is absent in the rigid HKUST‐1 and RhCu‐btc‐HKUST‐1 frameworks, underscoring the critical role of the incorporated defects in enabling this dynamic structural response (Figure 4e). This strategy thus enables the simultaneous incorporation of vacancies, mesoporosity, and free carboxylic acid groups within a single MOF architecture. The resulting defect‐rich, mesoporous framework displays breathing flexibility, a feature absent in the parent HKUST‐1 structure. Such a combination of properties provides a promising platform for applications in separation [46], catalysis [47], and proton conductivity [48].

## 3 Conclusion

In conclusion, we employed pre‐formed cages to template the synthesis of aperiodic, defect‐bearing networks related to HKUST‐1. The structure‐directing role of the persistent cavity enables the organised positioning of metal ions and linkers bearing missing coordination groups, thereby inducing localised Cu(II) paddlewheel vacancies within the framework. Because these defects can be arranged within the Cu(II)‐based cuboctahedral cavities of the framework in nine distinct configurations and because these cavities are structurally independent, the resulting materials display intrinsic aperiodicity. Furthermore, their higher defect density and reduced connectivity compared to HKUST‐1 impart combined mesoporosity and guest‐responsive flexibility. Overall, this approach provides a powerful route for designing complex MOFs with controlled aperiodicity that integrate diverse components and defect motifs.

## Author Contributions

**Francisco Sánchez‐Férez**: conceptualization, methodology, validation, investigation, formal analysis, writing – original draft. **Akim Khobotov‐Bakishev**: investigation, validation, methodology, conceptualization, writing – original draft. **Borja Ortín‐Rubio**: investigation, validation, methodology, writing – original draft. **Cornelia von Baeckman**: investigation, validation. **Laura Hernández‐López**: investigation, validation. **Alba Cortés‐Martínez**: investigation, validation. **Robert A. I. Paraoan**: investigation, validation. **Roberto Boada**: investigation, validation. **Judith Juanhuix**: investigation. **Felipe Gándara**: conceptualization, investigation, methodology, validation, writing – review and editing. **Andrew L. Goodwin**: investigation, writing – review and editing, methodology, formal analysis, supervision. **Arnau Carné‐Sánchez**: conceptualization, investigation, funding acquisition, methodology, validation, writing – review and editing, supervision. **Daniel Maspoch**: conceptualization, funding acquisition, validation, writing – review and editing, supervision, project administration.

## Conflicts of Interest

The authors declare no conflicts of interest.

## Supporting information




**Supporting File 1**: anie73336‐sup‐0001‐SuppMat.pdf.

**Supporting File 2**: anie73336‐sup‐0002‐DataFile.zip.

## Data Availability

The data that support the findings of this study are available from the corresponding author upon reasonable request.
