# Supplementary material for: Defect‐Guided Assembly of Aperiodic and Flexible Metal–Organic Frameworks From Pre‐Formed Cages
Source: Angew Chem Int Ed Engl. 2026 Jun 26;65(35):e9270871. doi: 10.1002/anie.9270871 (PMC13502634; doi:10.1002/anie.9270871)
Supplement: Supplementary file 1 [file ANIE-65-e9270871-s002.pdf]

Supporting Information

Defect-Guided Assembly of Aperiodic and Flexible Metal–Organic Frameworks from Pre-formed Cages

Francisco Sánchez-Férez,[a,b,+] Akim Khobotov-Bakishev,[a,b,+] Borja Ortín-Rubio,[a,b] Cornelia von Baeckman,[a,b] Laura Hernández-López,[a,b] Alba Cortés-Martínez,[a,b] Robert A. I. Paraoan,[d] Roberto Boada,[b] Judith Juanhuix,[e] Felipe Gándara,*[c] Andrew L. Goodwin,[d] Arnau Carné-Sánchez,*[a,b] and Daniel Maspoch*[a,b,f]

S1. Materials and methods

S1.1 Materials and characterisation

Rhodium acetate was purchased from Across Organics. 1,3-Benzenedicarboxylic acid (bdc), 1,4-benzenedicarboxylic acid (1,4-bdc), sodium carbonate, 1,3,5-benzenetricarbonyl trichloride, 2-(trimethylsilyl)ethanol, tetrabutylammonium fluoride (1.0 M in tetrahydrofuran (THF)), sodium hydroxide, and copper(II) nitrate trihydrate were purchased from Sigma-Aldrich. 2,5-furan-dicarboxylic acid (fdc) was purchased from Abcr. Solvents at HPLC grade were purchased from Fischer Chemicals. Deuterated solvents were purchased from Eurisotop. All reagents and solvents were used without further purification. Deionised water was obtained with a Milli-Q® system (18.2 MΩ·cm).

Field-emission scanning electron microscopy (FESEM) was performed in an FEI Quanta 650FEG ESEM and an FEI Magellan 400L XHR SEM. The average size distribution of each synthesised HKUST-1 analogue was statistically estimated from FESEM images by counting the sizes of 150 particles at different areas from each sample.

Transmission electron microscopy-energy Dispersive X-ray spectroscopy (TEM-EDX) was performed in an FEI Tecnai G2 F20 S-TWIN HRSTEM.

Fourier Transform Infrared Spectroscopy (FTIR-ATR) measurements were recorded on a PerkinElmer spectrometer, equipped with a universal attenuated total reflectance (ATR) accessory with a diamond window in the range 4000−400 cm^{-1}.

Proton nuclear magnetic resonance (^{1}H-NMR) spectra were acquired in a Bruker Avance NEO 400 MHz NMR spectrometer. All chemical shifts (δ) are given in ppm.

Synchrotron powder X-ray diffraction (PXRD) samples were collected at the XALOC-BL13 beamline of the ALBA Synchrotron Facility.[1] Samples were mounted into capillaries using colloidal suspensions. The experiments were carried out in transmission mode using an X-ray beam with a wavelength of λ = 0.82653 Å, directing the beam at the bottom of the capillary, with higher density of nanoparticles. Data was collected using a photon-counting DECTRIS PILATUS3 6M pixel-array detector. The diffraction patterns were acquired while rotating the capillary by 180º for typically 10 seconds. The acquisition time and the transmission were adjusted for each sample to maximize the counts without saturating the detector. The diffraction patterns were radially integrated using the FIT2D Program.[2]

Rietveld refinement was carried out with the use of the Reflex Module of Materials Studio. PXRD data was collected at the same beamline XALOC-BL13 beamline of the Alba Synchrotron Facility, in transmission mode, with a wavelength of 0.97912 Å to optimize the data in the low-q end of the diffraction profile, using the same experimental protocol as described above. A Thompson-Cox-Hastings function was used for profile description, with a 20-coefficient polynomial order background, and refinement of crystallite size for peak broadening description. The three representative cifs (named Periodic_Square, Periodic_Tetrahedral, and Aperiodic) are provided as part of the SI.

Defective RhCu-bdc-HKUST-1 supercell model generation and diffraction simulation. Three sets of four 4x4x4 supercell models of RhCu-bdc-HKUST-1 were made which incorporate different combinations of the nine Cu(II) cuboctahedral defect configurations. The first set includes all nine defect types, the second square-like only and the last tetrahedral-like defects. The cifs for the unit cells associated with each defect configuration from the Rietveld refinements had their lattice parameters adjusted to those of the high symmetry aperiodic model, while keeping fractional coordinates unchanged, in order to enable construction of supercells by stacking these cells in 3D space. Given the structural independence of these Cu(II) cavities within the extended framework, these supercell models were constructed as cifs by randomly placing one of the allowed defects for the given set with equal probability at each Cu(II) cuboctahedral site in the supercell. Three representative cifs (named HKUST4x4x4SqP, HKUST4x4x4Td and HKUST4x4x4mixed) are provided as part of the SI.

The PXRD pattern of each supercell configuration was simulated using CrystalDiffract® [CrystalMaker Software Ltd, Oxford, England (www.crystalmaker.com/crystaldiffract/)] using a wavelength of 1.5405 Å and Gaussian peaks of width 0.2°. The intensity profiles were extracted, convolved with Gaussian function of width 0.2° and the resulting profiles averaged across all four members of a given set. The 2θ angles of the intensity profile for each set were then scaled to match the synchrotron wavelength used in the experiments (0.9791 Å) and a Debye Waller factor of 0.2 $Å^2$ was applied, resulting in the powder patterns shown in Figure 2d.

The single crystal diffraction pattern of each supercell configuration was simulated using SingleCrystal® [CrystalMaker Software Ltd, Oxford, England (www.crystalmaker.com/singlecrystal/] using a wavelength of 1.5405 Å, Cam length of 0.05 m and spot size 0.0175 $Å^{-1}$. Laue symmetry averaging was applied as well as averaging across the patterns of all configurations in a given set. The hhl slices for each set as well as the average structure are shown in Figure 1c. Subtle differences in the forms of the diffuse scattering were found between the tetrahedral-like, square-like and mixed models.

Thermogravimetric analysis (TGA) curves were carried out in a Pyris Perkin Elmer TGA 8000 under nitrogen atmosphere and a heating rate of 10 °C/min.

Volumetric N_2 isotherms were collected at 77 K using a High-Resolution ASAP 2460 (Micromeritics). The temperature for N_2 adsorption measurements was controlled by using a liquid nitrogen bath. BET surface values were calculated according to the BETSI method.[3] Pore size distribution (PSD) was estimated using a density functional theory (DFT) model (N_2 – cylindrical pores – oxide surface) implemented in the Microactive 4.06 software with a regularisation factor of 0.01.

Gravimetric MeOH-sorption isotherms were measured using a Surface Measurement System Ltd instrument (DVS vacuum). The initial weight of the activated sample (≈10 mg) was constantly monitored with a high-resolution microbalance (±0.1 µg) and recorded at 298 K (±0.2 K) while exposed to MeOH vapor across the range of vapor pressures.

Inductively coupled plasma mass spectrometry (ICP-MS) analyses were performed in an external company, Leitat, in an ICP-MS Agilent 7500. 10 mg of sample were digested with 4 mL of concentrated ultrapure nitric acid (HNO_3, 70%) in an analytical microwave Anton Paar MulCwave 7000 at 250 °C. Subsequently, the digestion residue obtained was suitably diluted with a nitric acid solution (HNO_3, 2%) to analyse the elements of interest by ICP-MS. The quantification was performed by interpolation on a calibration curve prepared from commercial standards of the elements of interest. The assays were performed in duplicates.

X-ray absorption spectroscopy (XAS) measurements were performed at the B18 beamline of the Diamond Light Source synchrotron facility (Didcot, United Kingdom).[4] The measurements were performed in transmission mode at room temperature using QEXAFS scanning mode and Si(111) monochromator crystals. Samples were loaded into borosilicate capillaries of 10 µm wall thickness (2mm diameter). XAS data analysis (i.e., spectra averaging, background substraction, and spectra normalisation) was performed according to standard procedures using the ATHENA program included in the Demeter software package.[5]

S1.2 Experimental Methods

S1.2.1 Synthesis of COOH-RhMOP, HKUST-1, and RhCu-btc-HKUST-1. COOH-RhMOP was synthesised according to a previously described methodology.[6] Cu-HKUST-1 and RhCu-btc-HKUST-1 were synthesised according to the previously described methodologies.[7,8]

S1.2.2 Synthesis of RhCu-bdc-HKUST-1 ($[Rh_{1.5}Cu(btc)_{1.5}(bdc)_{0.5}(H_2O)_3]$). A N,N-dimethylformamide (DMF) solution (0.5 mL) containing 5.16 mg (21.36 µmol) of copper nitrate trihydrate ($Cu(NO_3)_2{\cdot}3H_2O$) was added to a DMF solution (1 ml) containing 10 mg (1.34 µmol) of COOH-RhMOP. Next, a DMF solution (0.5 ml) containing 1.78 mg (10.71 µmol) of isophthalic acid (bdc) was added to the former solution. The resulting green mixture was heated at 100 °C for 24 h. The obtained suspension was centrifuged to obtain a green solid that was washed with DMF (x3), MeOH (x3), and dried at 85 °C for 1 hour. The green powder was further washed with MeOH (x3), water (x5), acetone (x2), and then either resuspended in MeOH to obtain a colloidal suspension or dried for further characterization. ICP-MS (Cu/Rh ratio): 0.69 ± 0.01.

S1.2.3 Synthesis of RhCu-fdc-HKUST-1 ($[Rh_{1.5}Cu(btc)_{1.5}(fdc)_{0.5}(H_2O)_3]$). 10 mg (1.34 µmol) of COOH-RhMOP were solubilised in a DMF solution (0.5 ml) containing 5.16 mg (21.36 µmol) of $Cu(NO_3)_2{\cdot}3H_2O$. Next, a DMF solution (0.5 ml) containing 1.67 mg (10.71 µmol) of 2,5-furandicarboxylic acid (fdc) was added to the former solution. The resulting green mixture was heated at 120 °C for 6 h. The obtained suspension was centrifuged to obtain a green solid that was washed with DMF (x3), MeOH (x3), and dried at 85 °C for 1 hour. The green powder was further washed with MeOH (x3), a water : MeOH mixture (1:1 (x5)), acetone (x2), and then either resuspended in MeOH to obtain a colloidal suspension or dried for further characterization. ICP-MS (Cu/Rh ratio): 0.71 ± 0.01.

S.1.2.4 Basic disassembly of RhCu-bdc-HKUST-1. In a typical experiment, 5 mg of RhCu-bdc-HKUST-1 were dispersed in 500 µL D_2O. Then, 5 µL of a 3M stock solution of sodium deuteroxide (NaOD) were added to this dispersion. The mixture was gently stirred until RhCu-bdc-HKUST-1 was completely dissolved forming a green solution. This solution was then analysed through ^{1}H-NMR to determine the COOH-RhMOP:bdc molar ratio.

S.1.2.5 Acidic disassembly of RhCu-fdc-HKUST-1. In a typical experiment, 5 mg of RhCu-fdc-HKUST-1 were dispersed in 500 µL DMF-d_7. Then, 5 µL of a 3M stock solution of deuterium chloride (DCl) were added to this dispersion. The mixture was gently stirred until RhCu-fdc-HKUST-1 was completely dissolved forming a green solution. This solution was then analysed through ^{1}H-NMR to determine the COOH-RhMOP:fdc molar ratio.

S.1.2.6 Study of the crystallinity recovery of RhCu-bdc-HKUST-1 and RhCu-fdc-HKUST-1. A 10 mg sample was dried under vacuum at 85 °C for 4 h to remove all guest solvent molecules, leading to amorphisation, as confirmed by PXRD. Subsequently, the samples were immersed for 2 days in the following solvents: water, MeOH, EtOH, THF, acetonitrile, acetone, chloroform, hexane, and toluene. The samples were then loaded into capillaries as colloidal suspensions in the corresponding solvent, and PXRD data were collected.

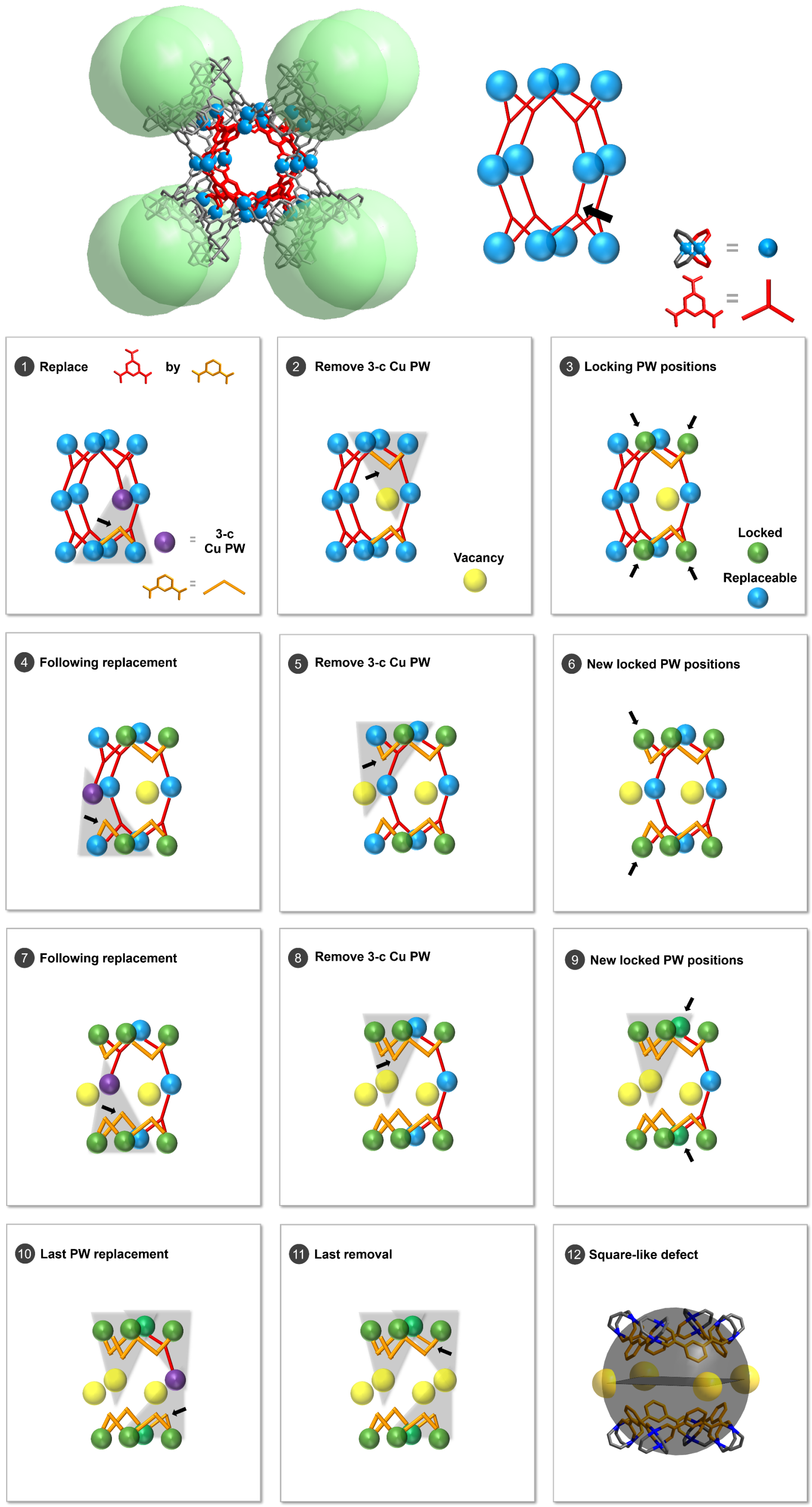

Figure S1. Representation of the structural analysis performed to identify the nine feasible defect configurations, illustrating one of the three square-like vacancy arrangement.

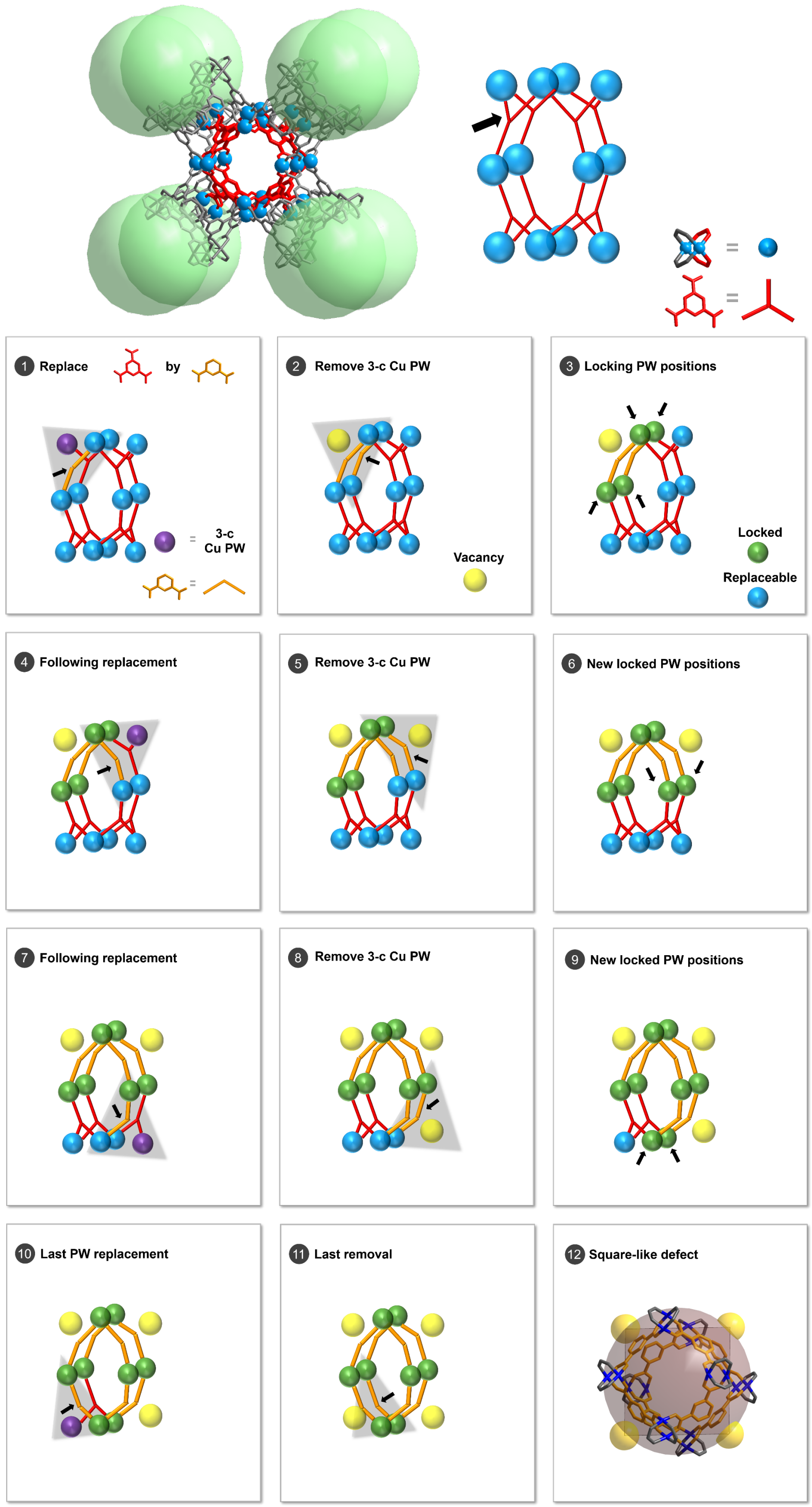

Figure S2. Representation of the structural analysis performed to identify the nine feasible defect configurations, illustrating one of the three square-like vacancy arrangement.

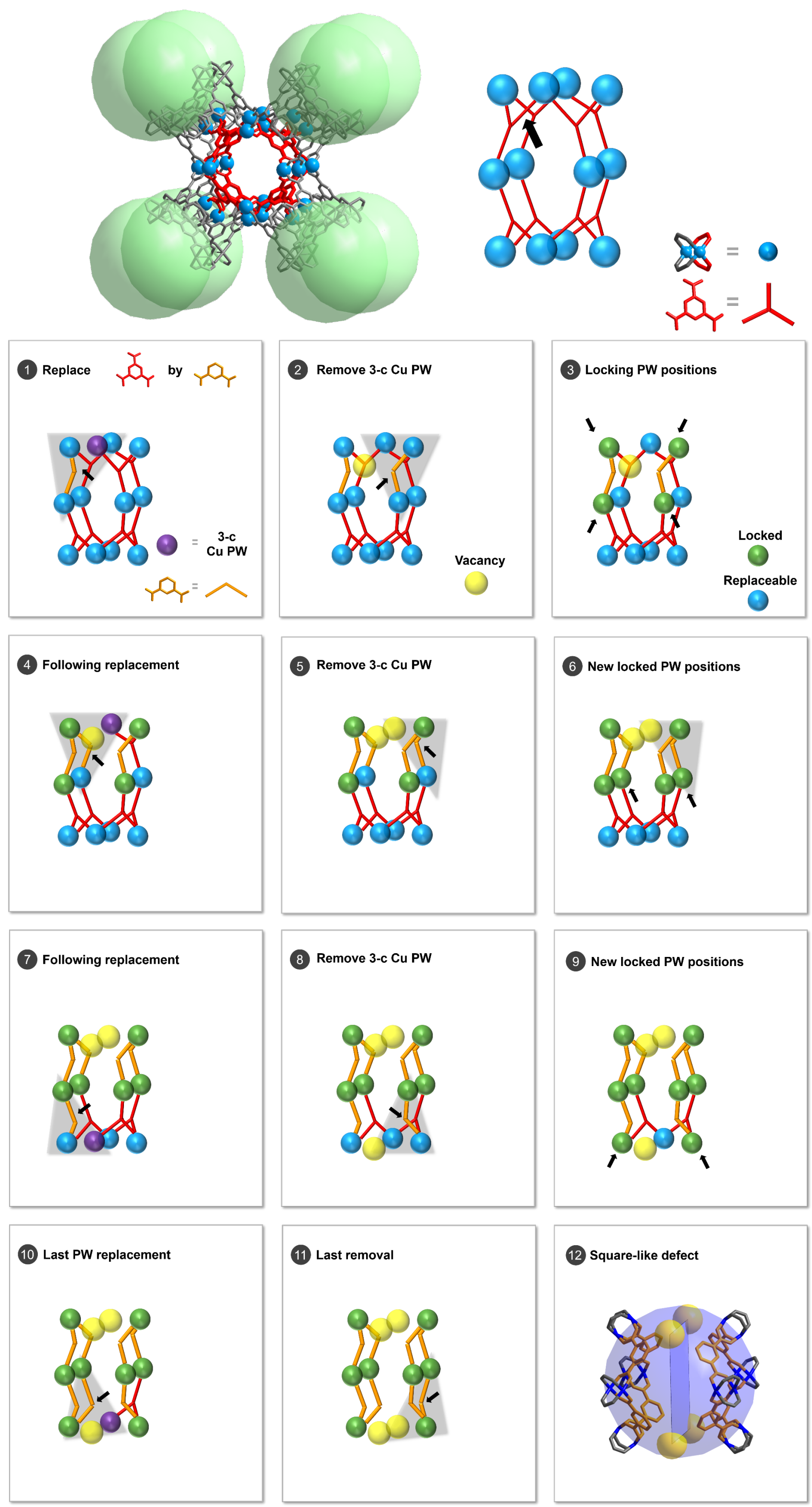

Figure S3. Representation of the structural analysis performed to identify the nine feasible defect configurations, illustrating one of the three square-like vacancy arrangement.

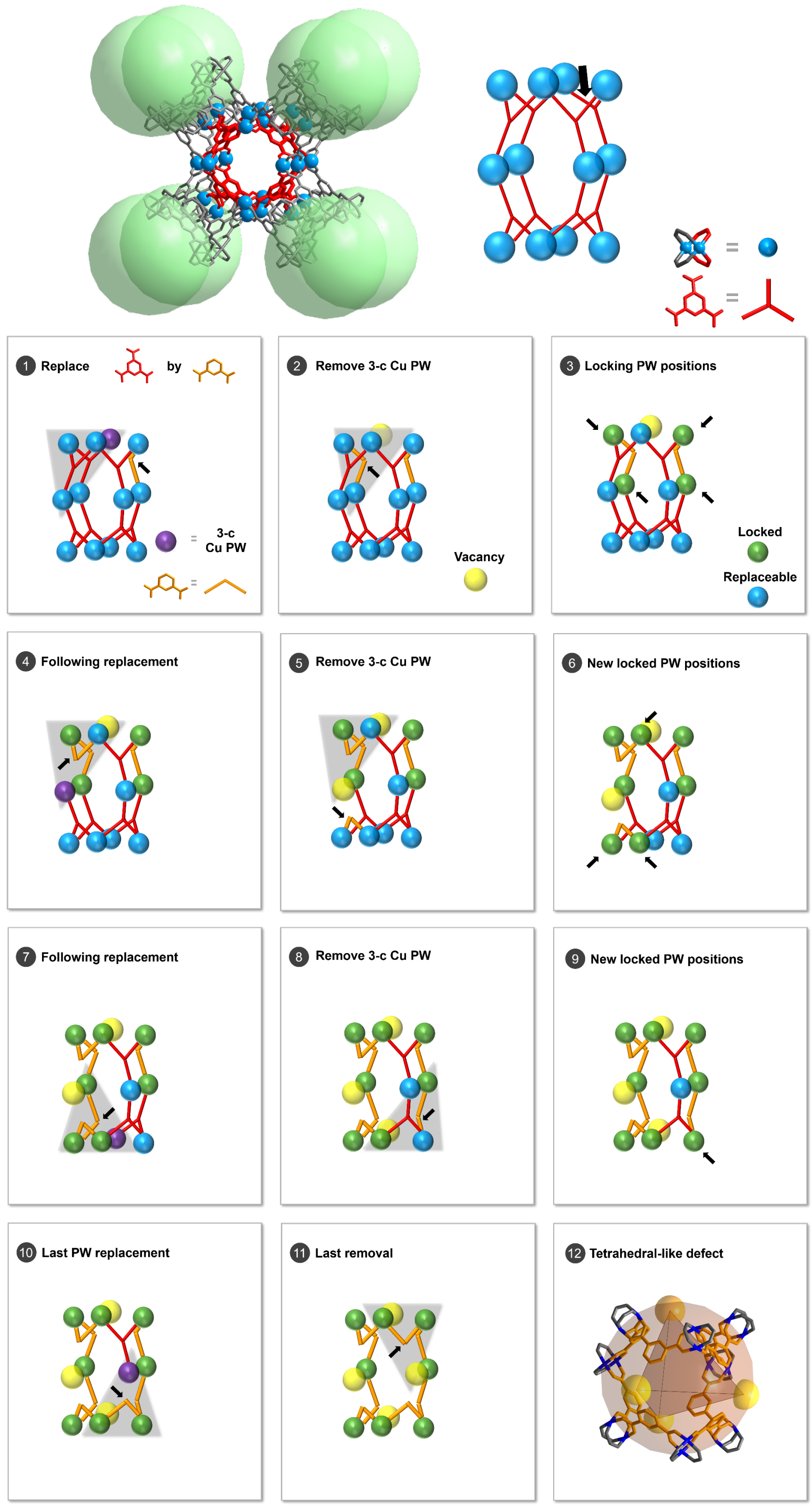

Figure S4. Representation of the structural analysis performed to identify the nine feasible defect configurations, illustrating one of the six tetrahedral-like vacancy arrangement.

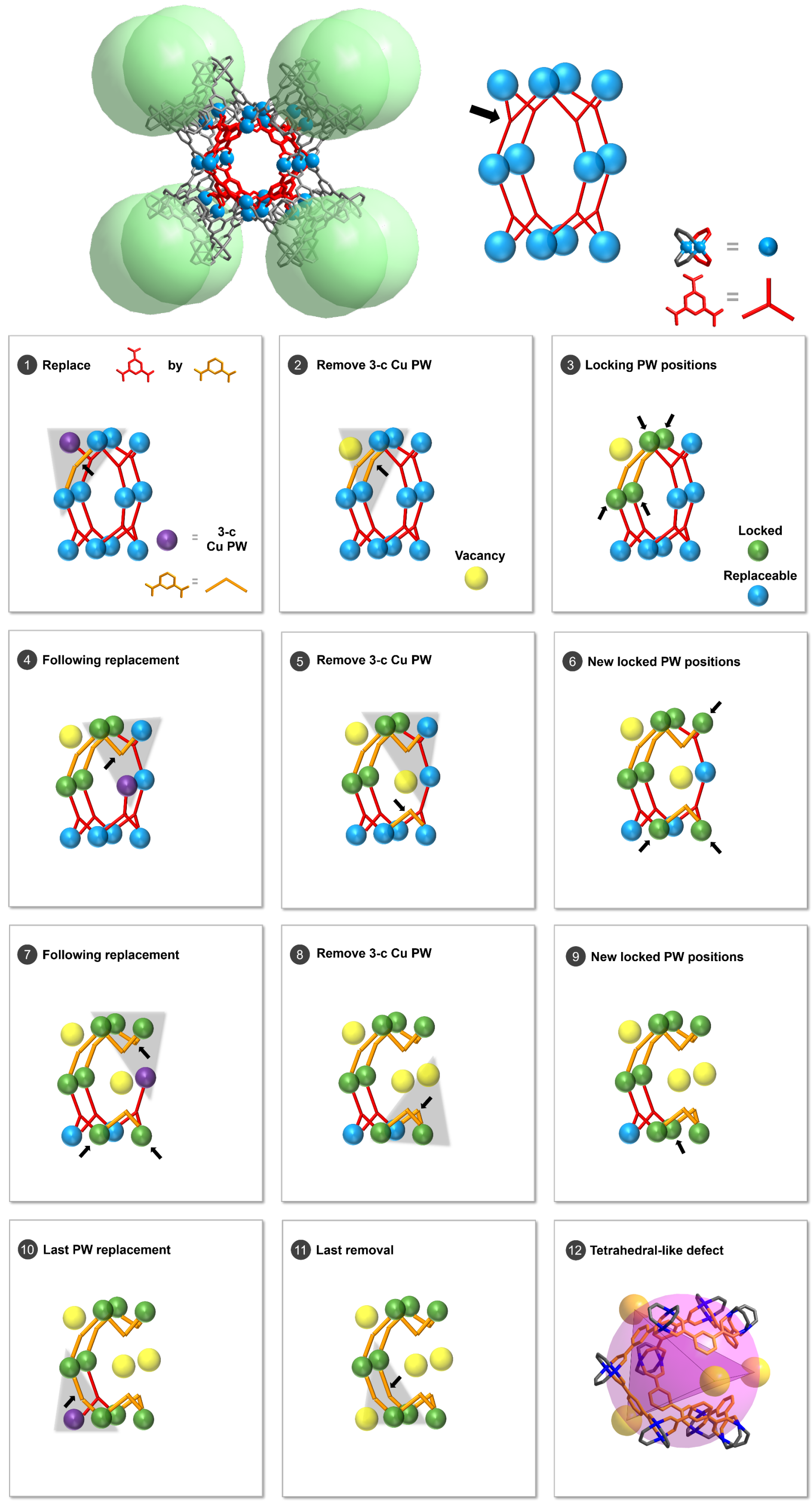

Figure S5. Representation of the structural analysis performed to identify the nine feasible defect configurations, illustrating one of the six tetrahedral-like vacancy arrangement.

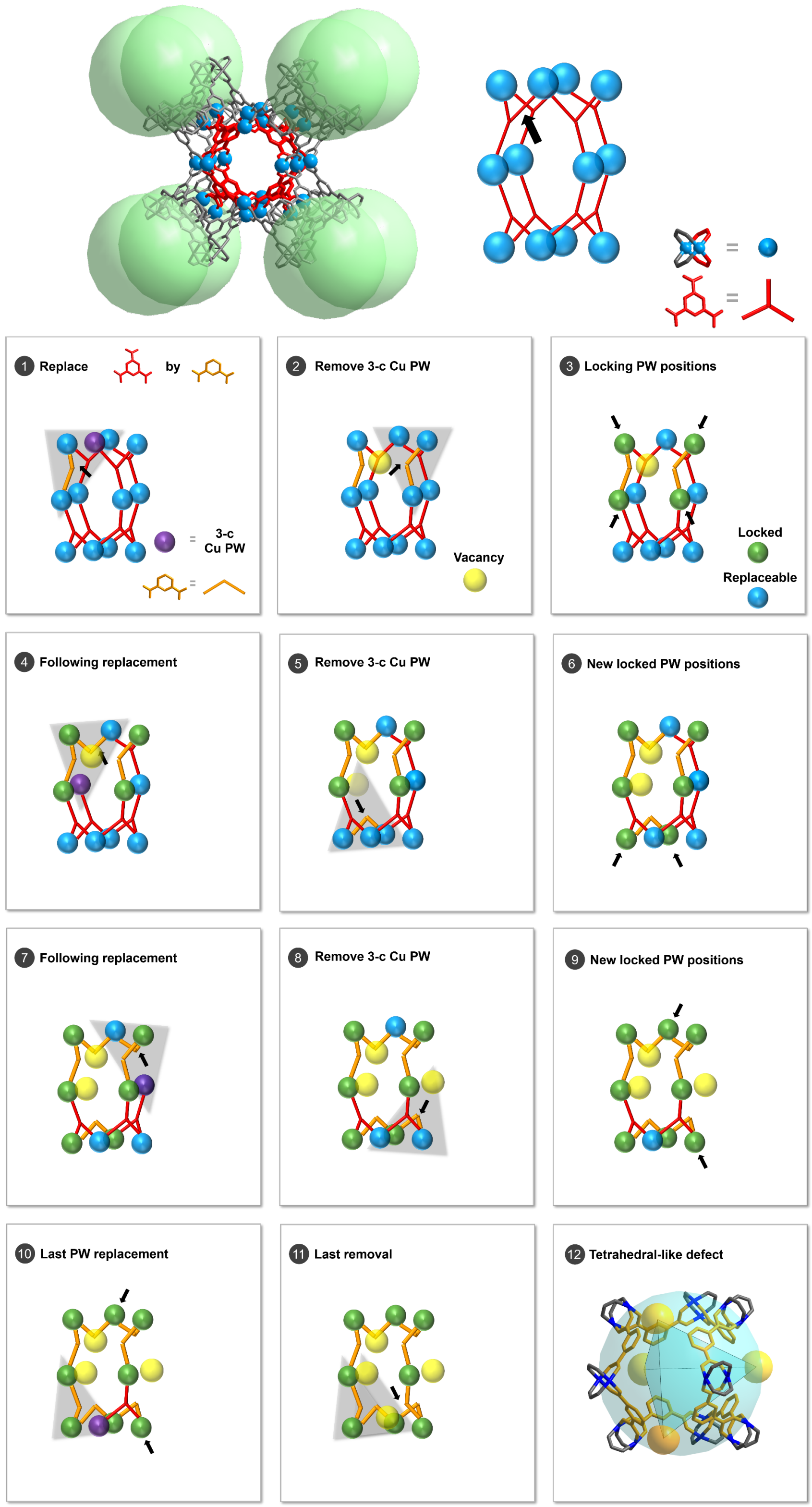

Figure S6. Representation of the structural analysis performed to identify the nine feasible defect configurations, illustrating one of the six tetrahedral-like vacancy arrangement.

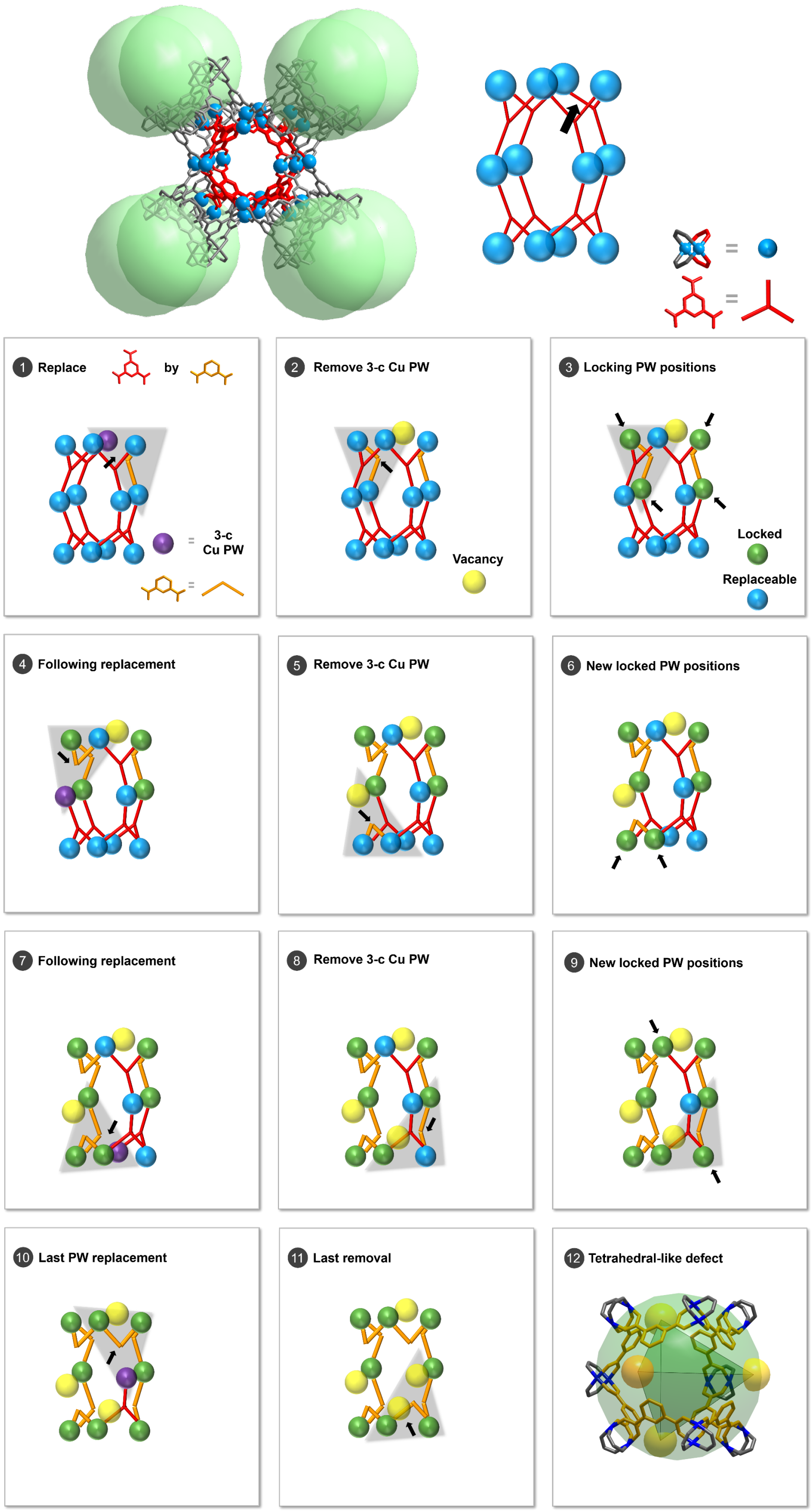

Figure S7. Representation of the structural analysis performed to identify the nine feasible defect configurations, illustrating one of the six tetrahedral-like vacancy arrangement.

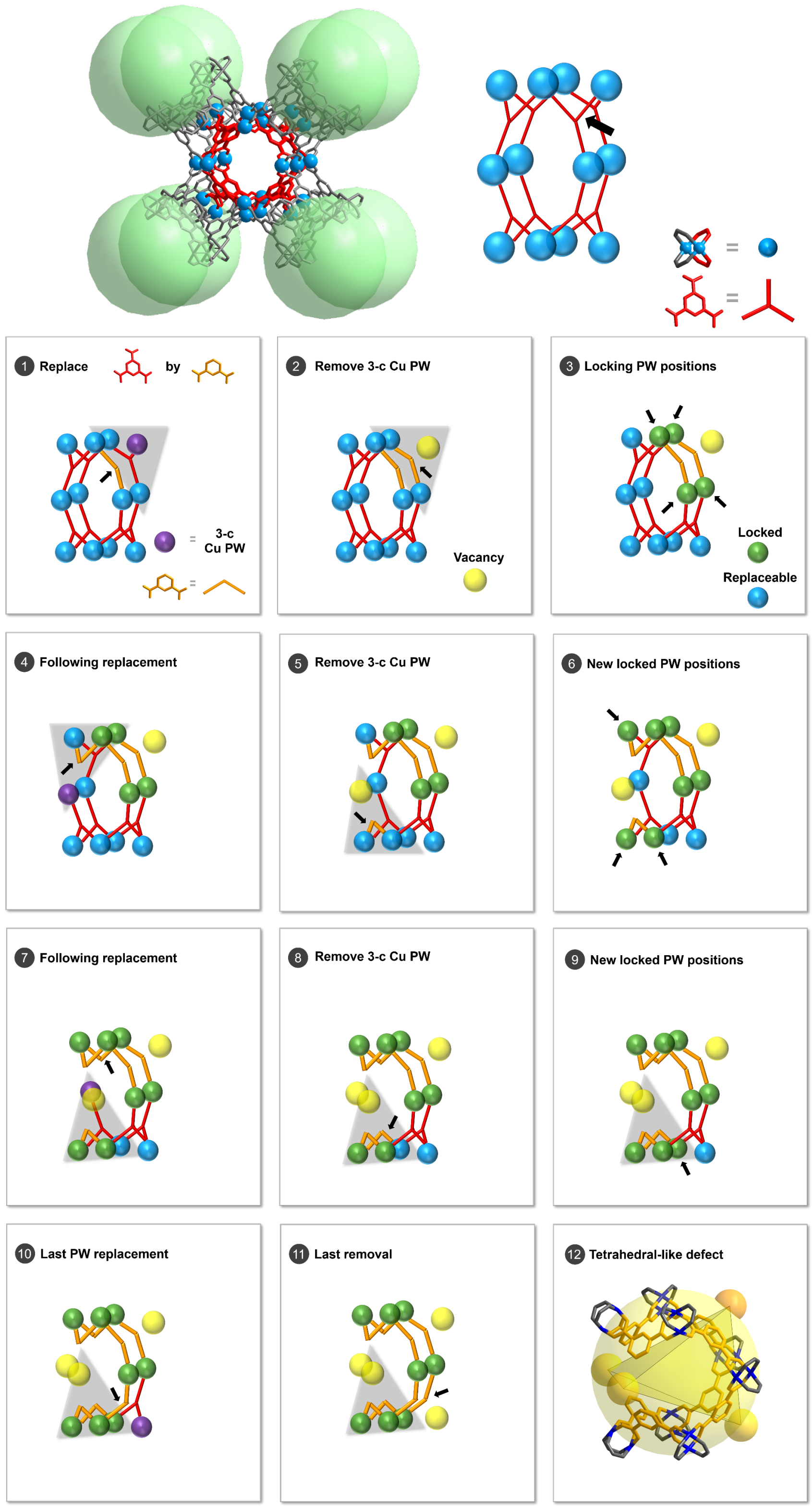

Figure S8. Representation of the structural analysis performed to identify the nine feasible defect configurations, illustrating one of the six tetrahedral-like vacancy arrangement.

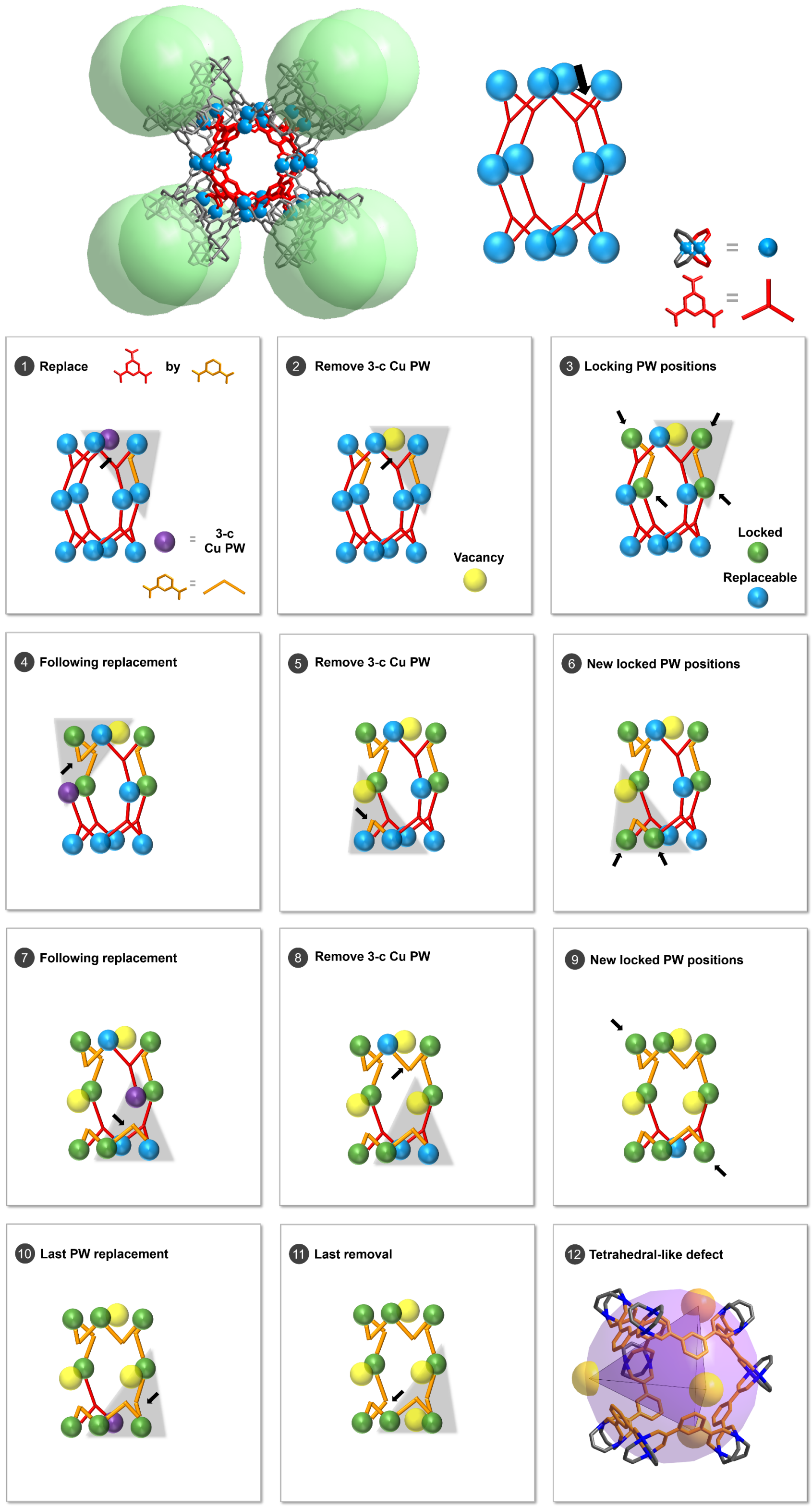

Figure S9. Representation of the structural analysis performed to identify the nine feasible defect configurations, illustrating one of the six tetrahedral-like vacancy arrangement.

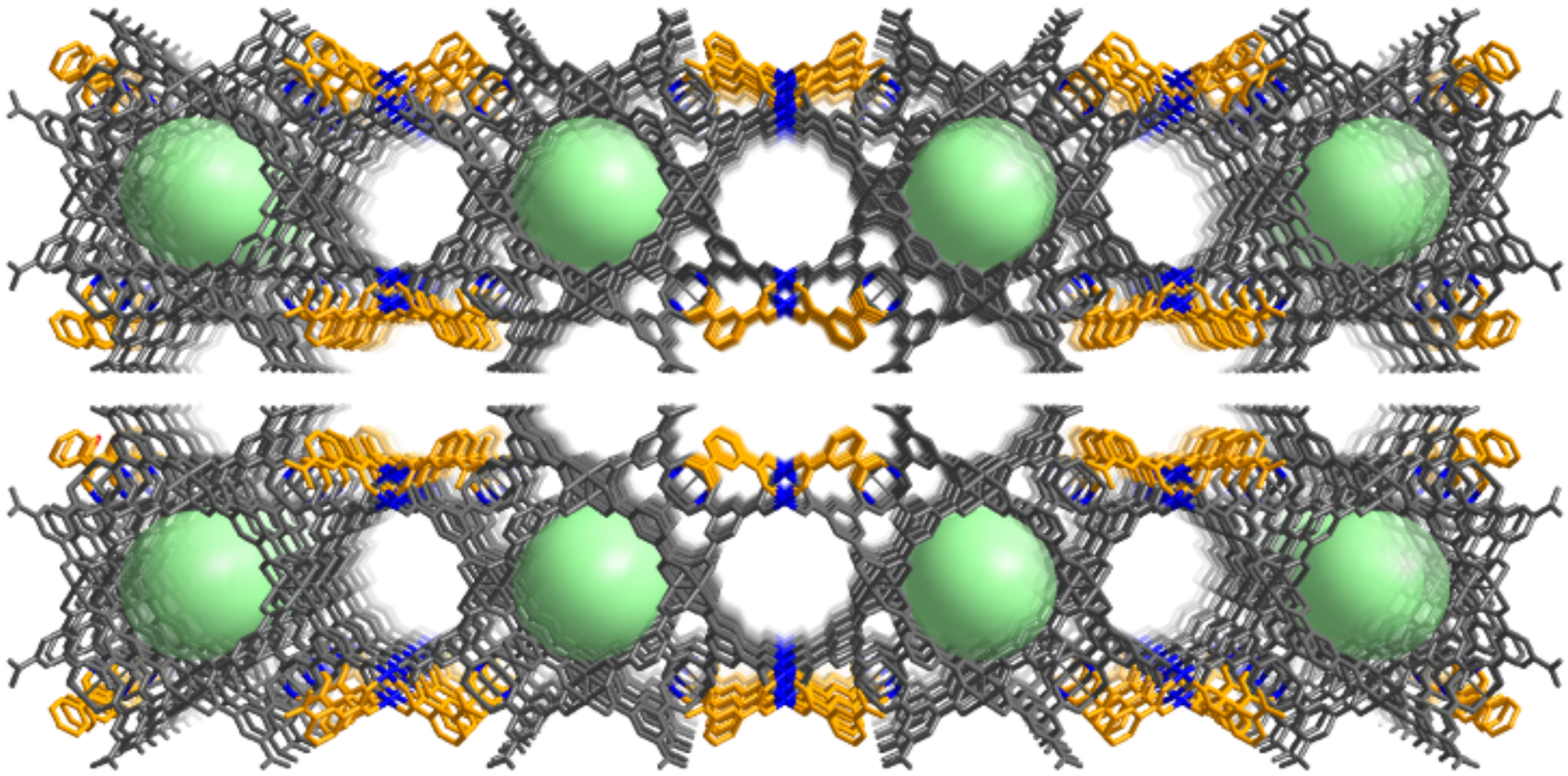

Figure S10. Representation of the 2D periodic tetragonal structure (space group *P4/mmm*) expected if all Cu(II)-based cuboctahedral cavities adopt the same square-like vacancy arrangement. The bdc ligands are highlighted in orange. View along a axis.

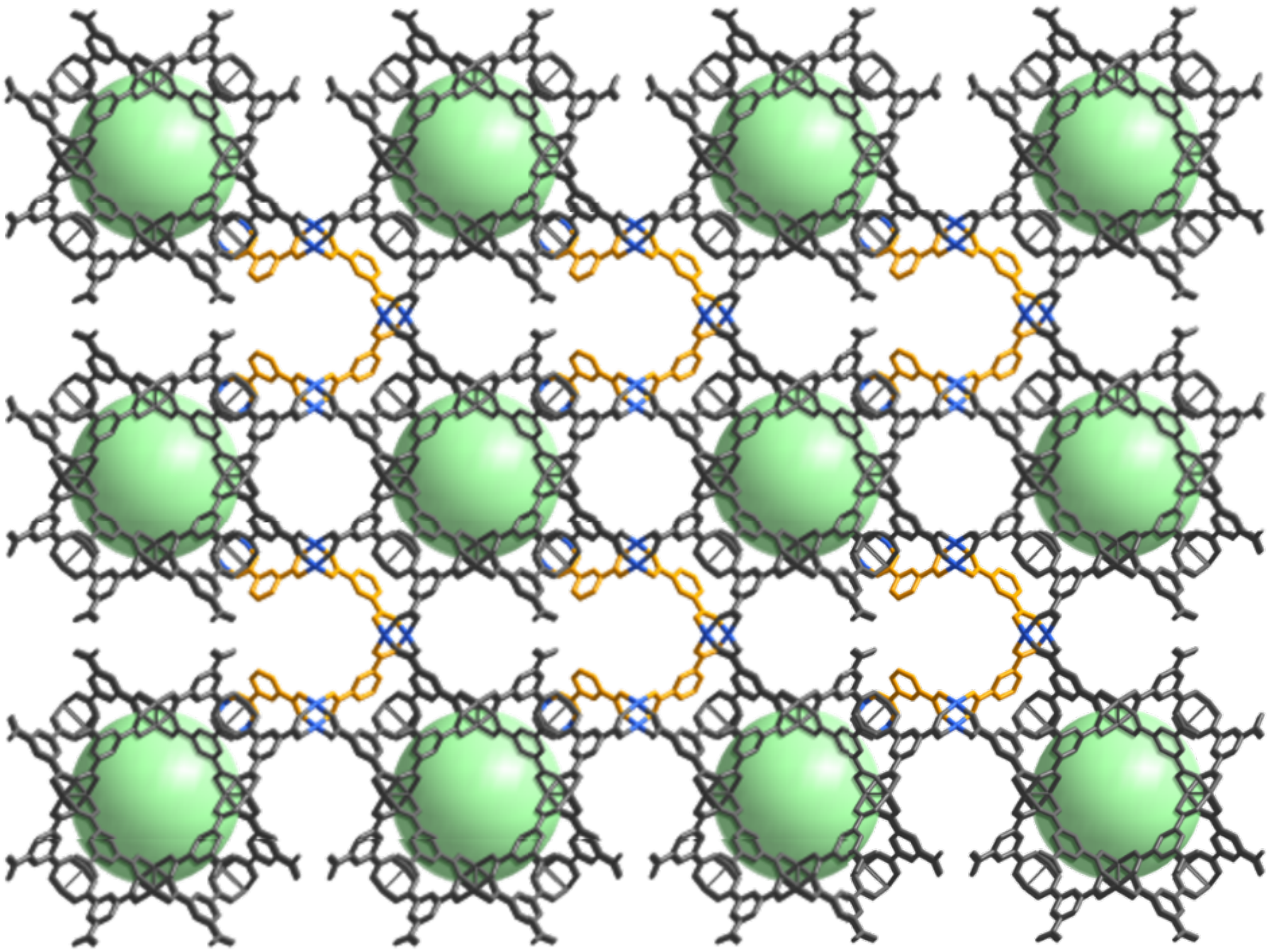

Figure S11. Representation of the 3D periodic structure (space group *P−4m2*) expected if all Cu(II)-based cuboctahedral cavities adopt the same tetrahedral-like vacancy arrangement. The bdc ligands are highlighted in orange. View along a axis.

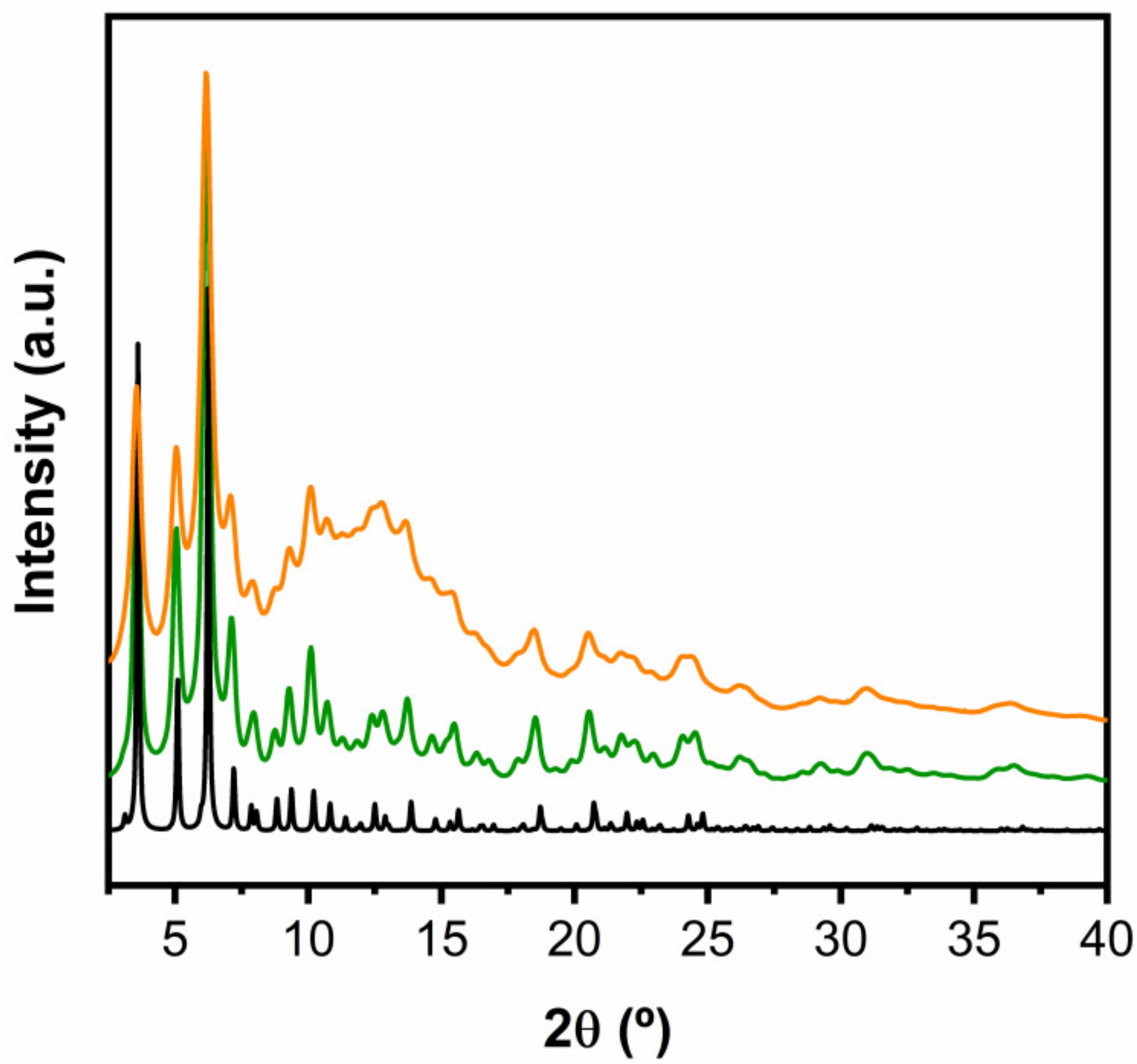

Figure S12. PXRD patterns of RhCu–bdc–HKUST-1 (orange) compared to RhCu–btc–HKUST-1 (green) and simulated HKUST-1 (black).

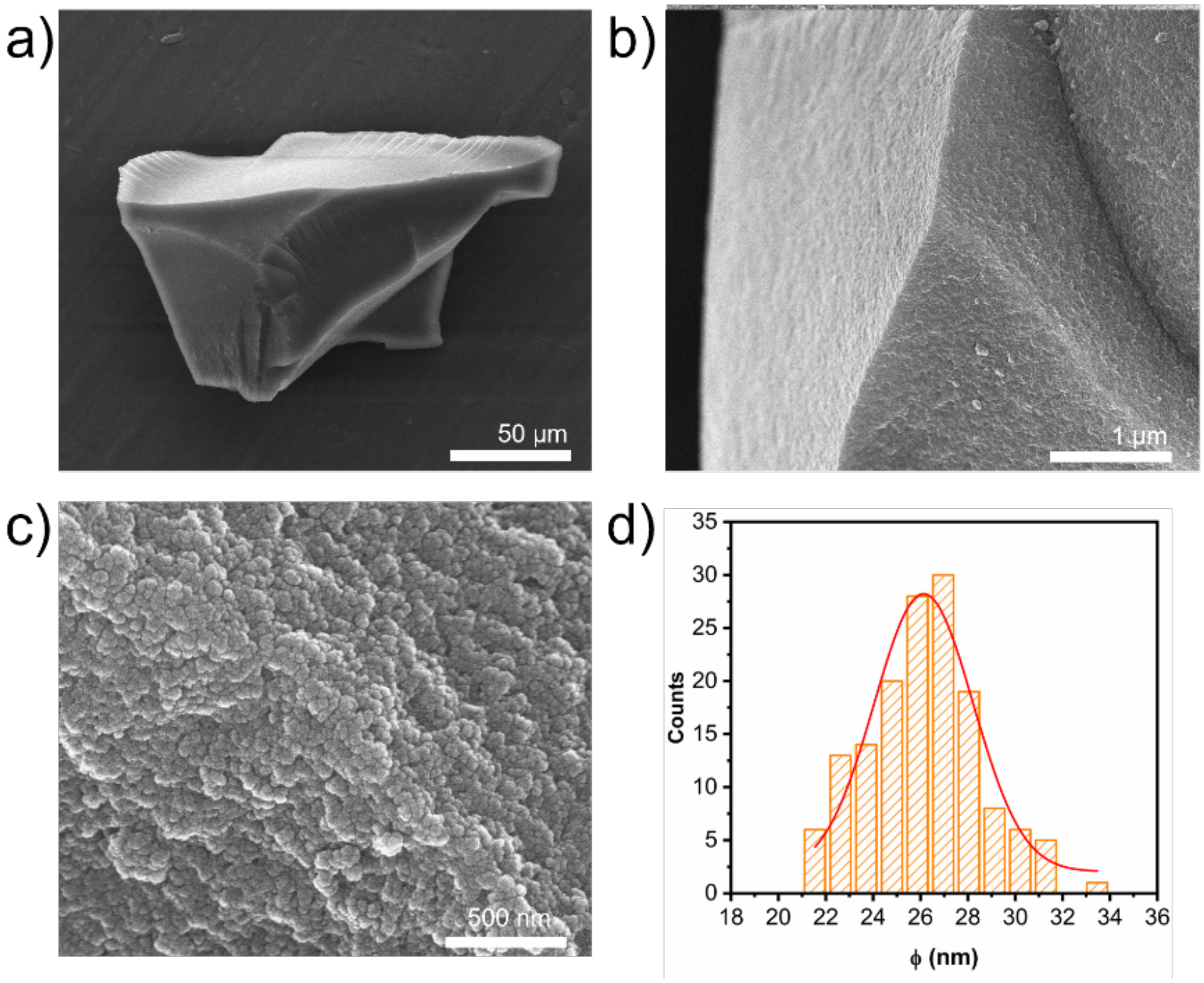

Figure S13. (a-c) Representative FESEM images of RhCu-bdc-HKUST-1, revealing the formation of monolithic polycrystalline aggregates (a, b) composed of nanocrystallites (c). These monoliths were formed upon drying the colloidal solution resulting from the reaction. (d) Particle size distribution histogram of RhCu-bdc-HKUST-1 nanocrystallites with a mean particle size of 26 ± 2 nm.

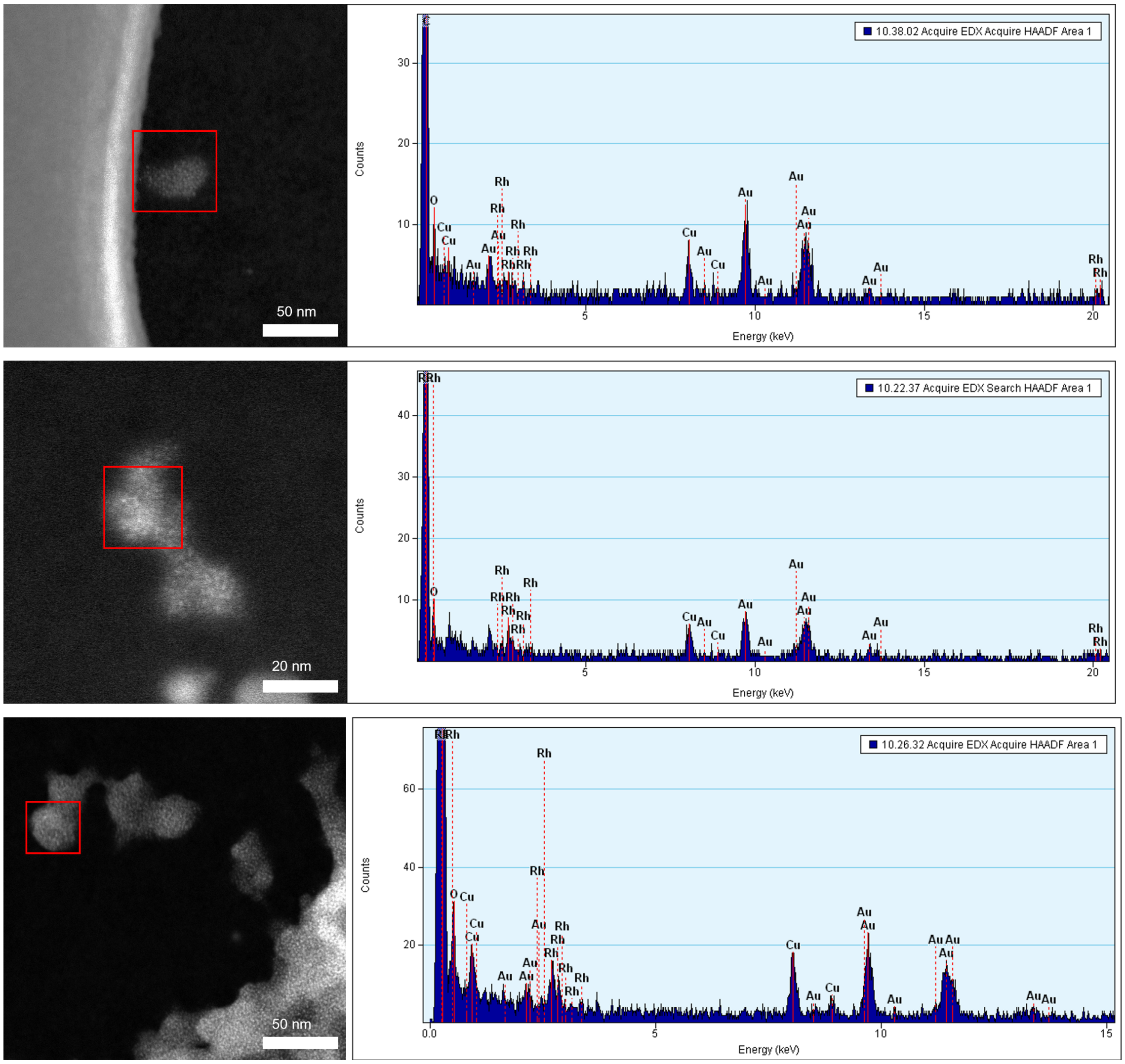

Figure S14. Representative high-angle annular dark-field scanning transmission electron microscopy (HAADF-STEM) images (left) and the corresponding EDX spectra (right) obtained from single RhCu-bdc-HKUST-1 crystalline particles. Note that Au signals stem from the TEM grid used for the measurements.

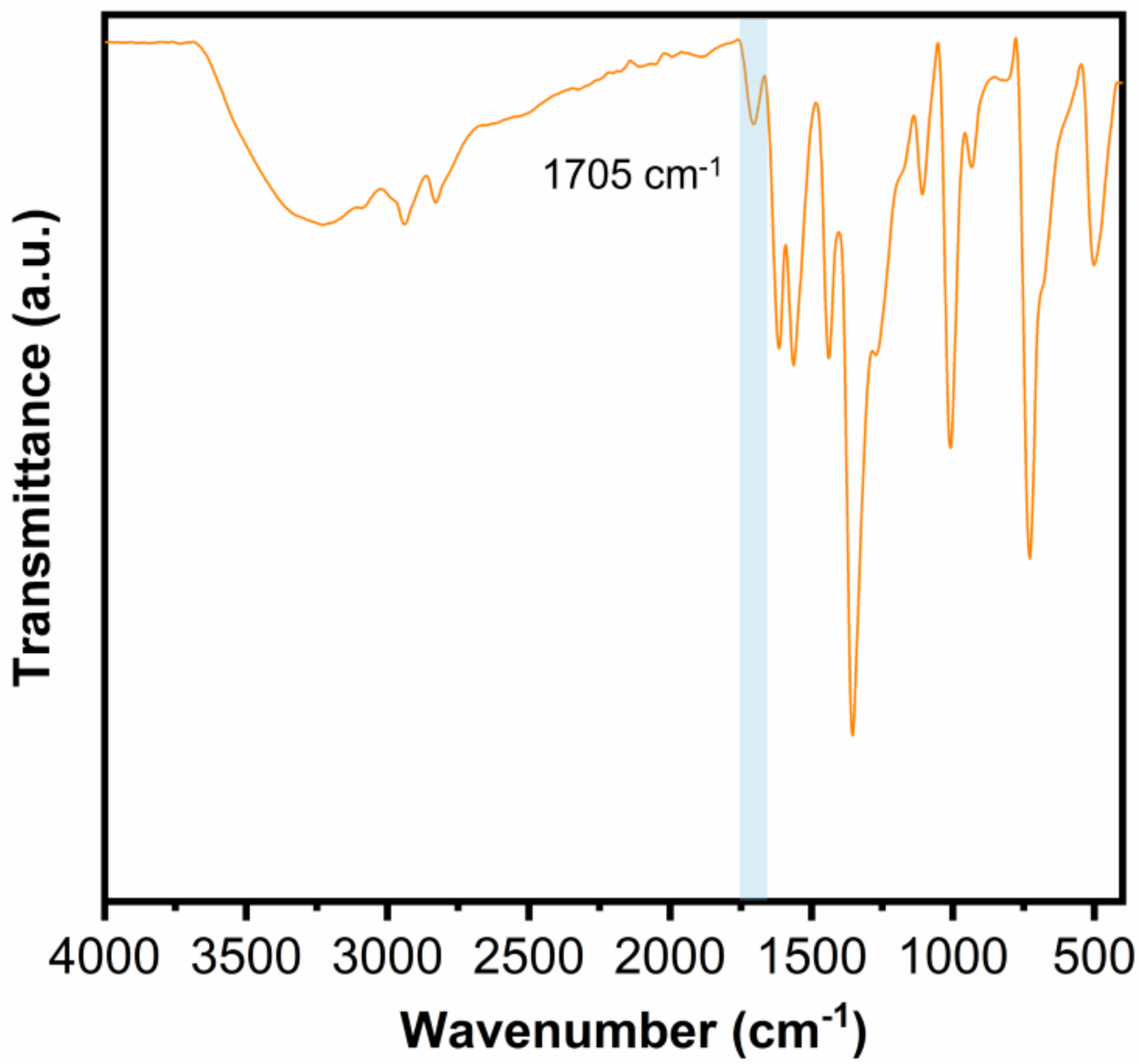

Figure S15. FTIR-ATR spectrum of RhCu-bdc-HKUST-1, highlighting the band at 1705 cm^{-1}.

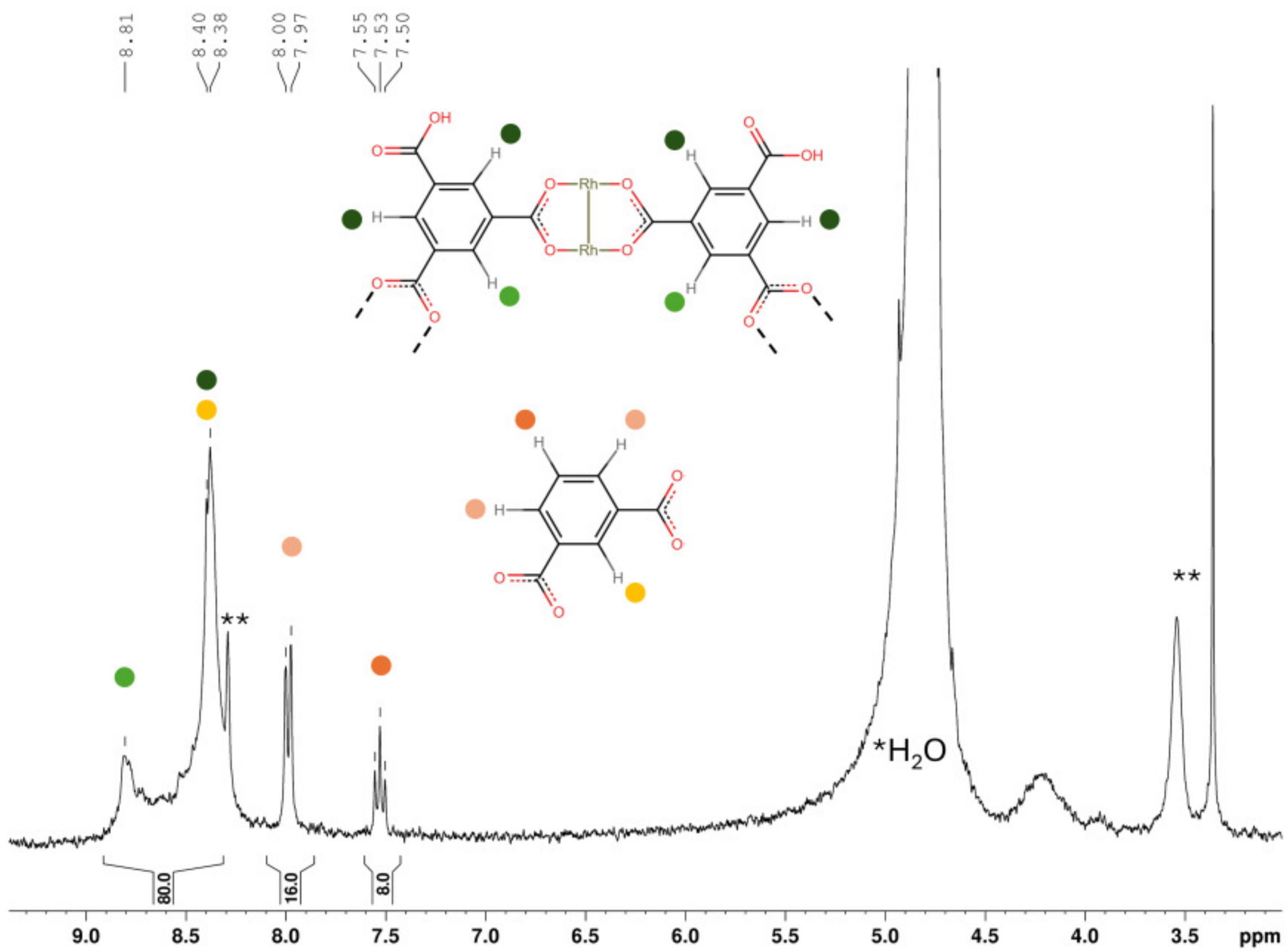

Figure S16. ^{1}H NMR spectrum (400 MHz, D_2O) of digested RhCu-bdc-HKUST-1, obtained after the addition of a NaOD solution under stirring. ** indicates DMF. Note that in the proposed structure of RhCu-bdc-HKUST-1 there are 8 bdc ligands per COOH-RhMOP and 72 btc protons in each COOH-RhMOP. The observed integration ratios are in agreement with the expected formula.

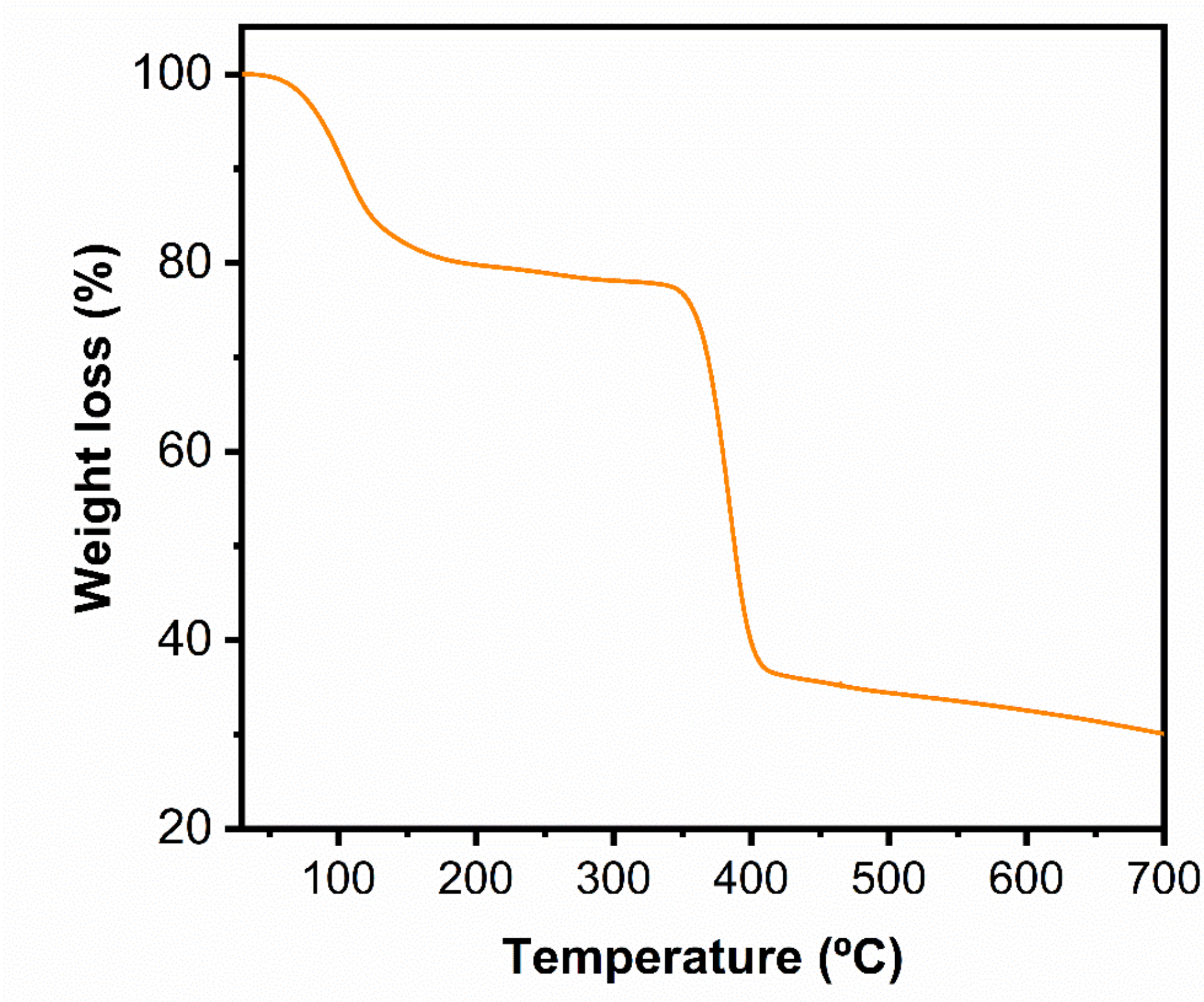

Figure S17. Thermogravimetric analysis (TGA) of RhCu-bdc-HKUST-1. Note that the weight loss up to 200 ºC is associated to the remaining solvent molecules coordinated to the metal sites. The decomposition temperature of RhCu-bdc-HKUST-1 (340 ºC) is in good agreement to reported values for the parent HKUST-1 and RhCu-btc-HKUST-1.[5,6]

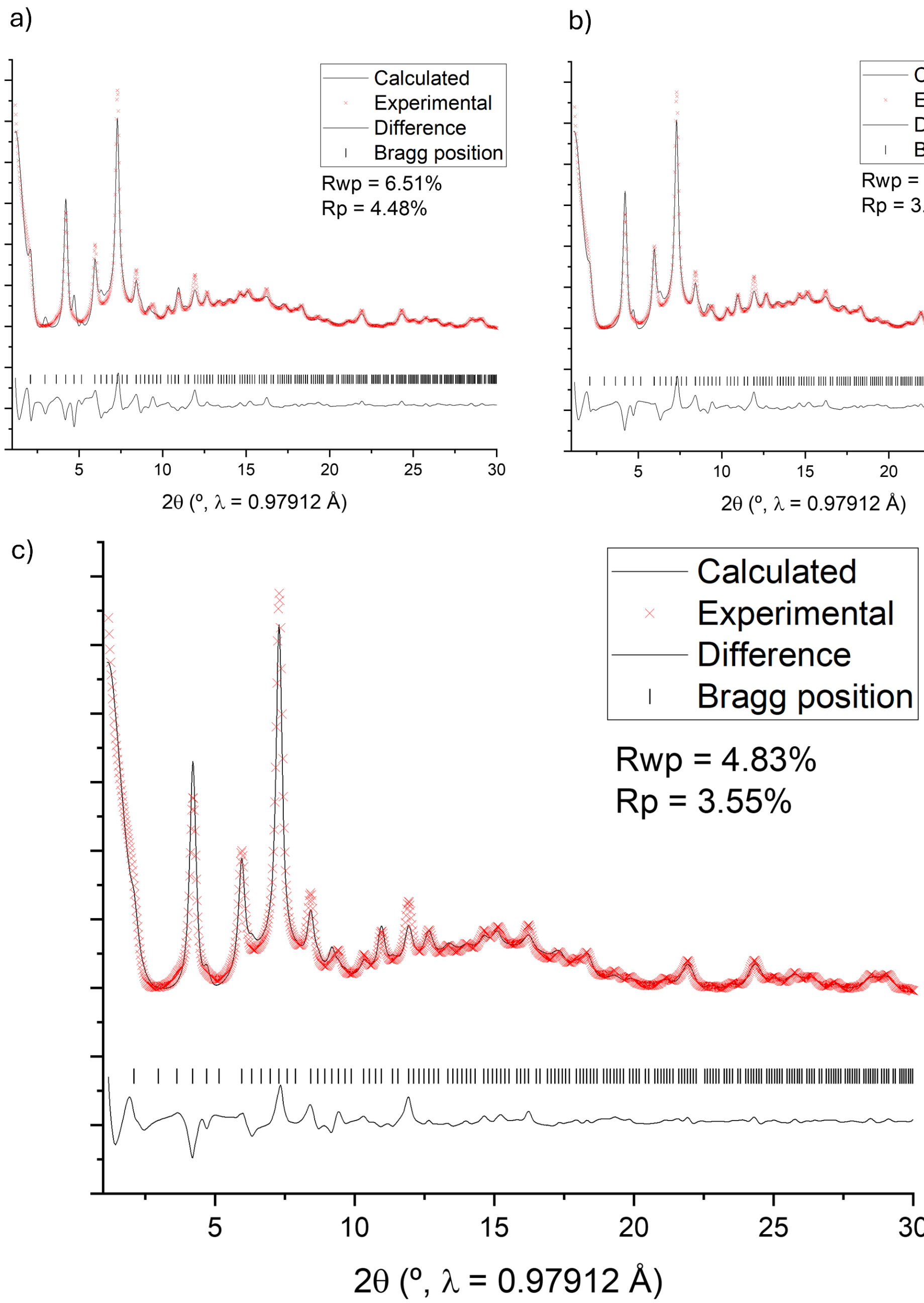

Figure S18. (a-c) Rietveld refinement analysis of RhCu-bdc-HKUST-1 for the two periodic 2D and 3D structures with space group *P4/mmm* (a) and with space group *P*−4*m*2 (b), and the aperiodic model with space group *Pm*−3*m* (c), obtaining R*wp* = 6.51%, 5.38%, and 4.83%, respectively.

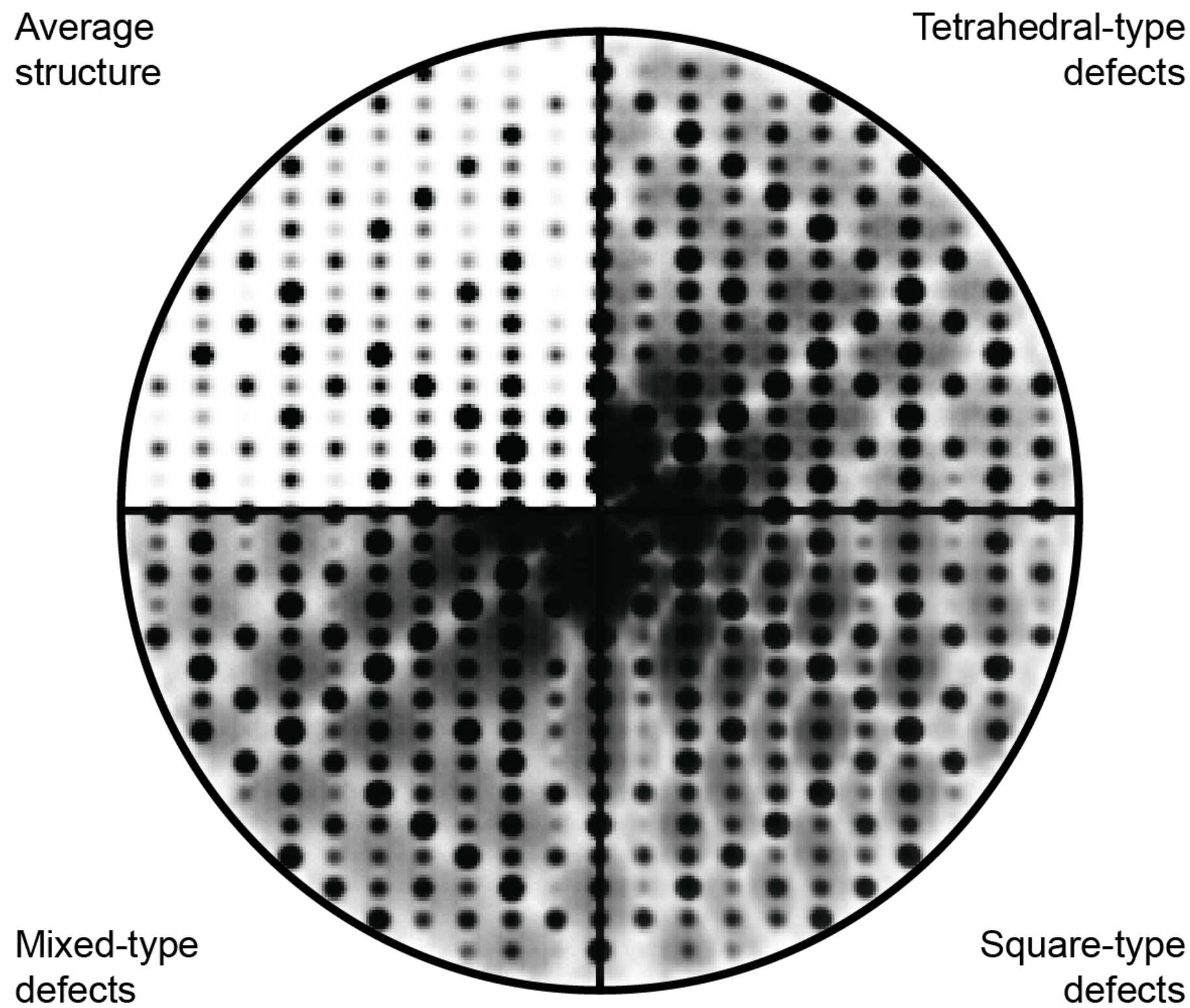

Figure S19. Single-crystal X-ray diffraction patterns (hhl slice) calculated from atomistic models of RhCu-bdc-HKUST-1 with different paddlewheel vacancy distributions. Note that the shape of the weak diffuse scattering features differs for tetrahedral-type and square-type defects.

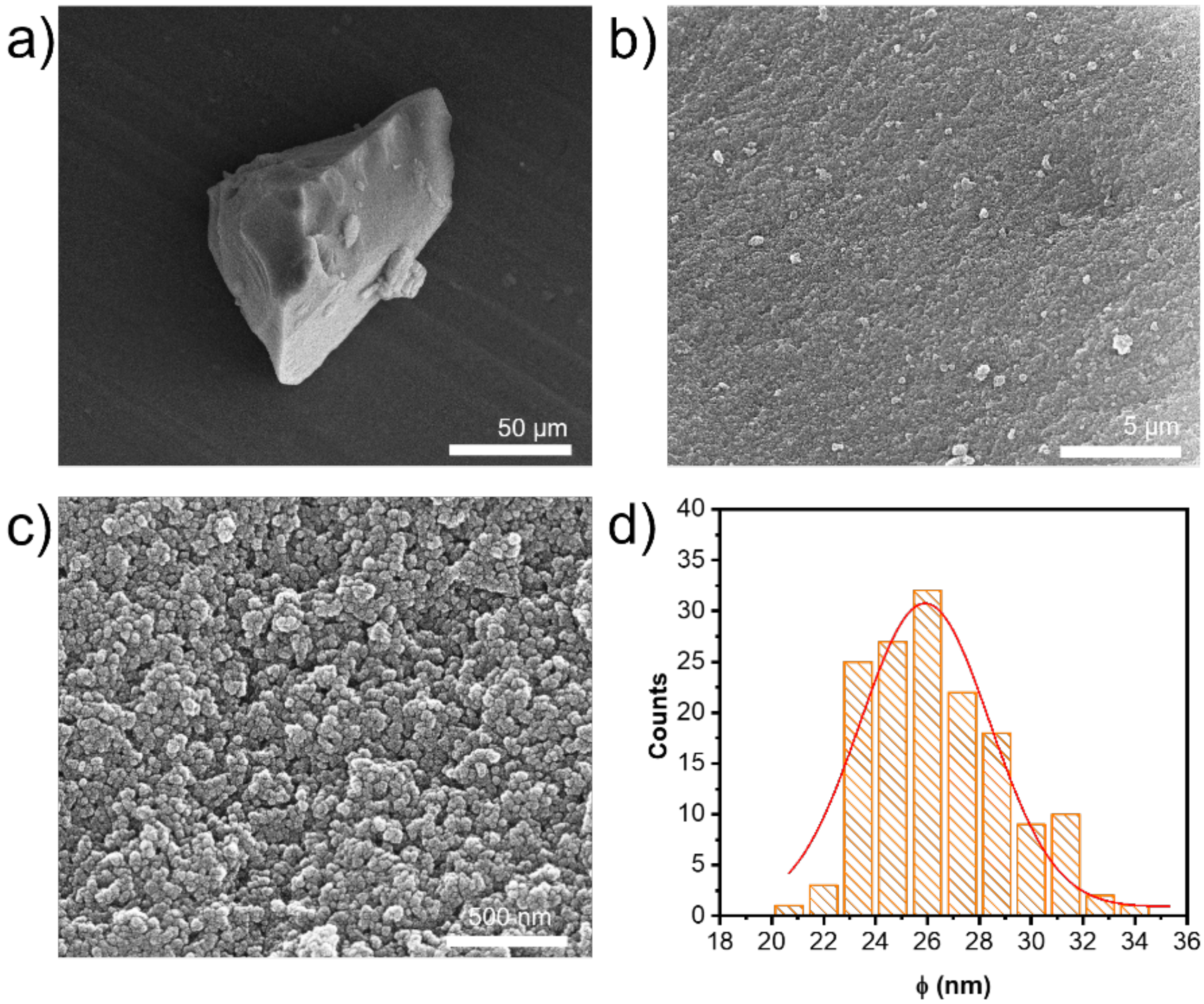

Figure S20. (a-c) Representative FESEM images of RhCu-fdc-HKUST-1, revealing the formation of monolithic polycrystalline aggregates composed of nanocrystallites. These monoliths were formed upon drying the colloidal solution resulting from the reaction. (d) Particle size distribution histogram of RhCu-fdc-HKUST-1 nanocrystallites with a mean particle size of 26 ± 3 nm.

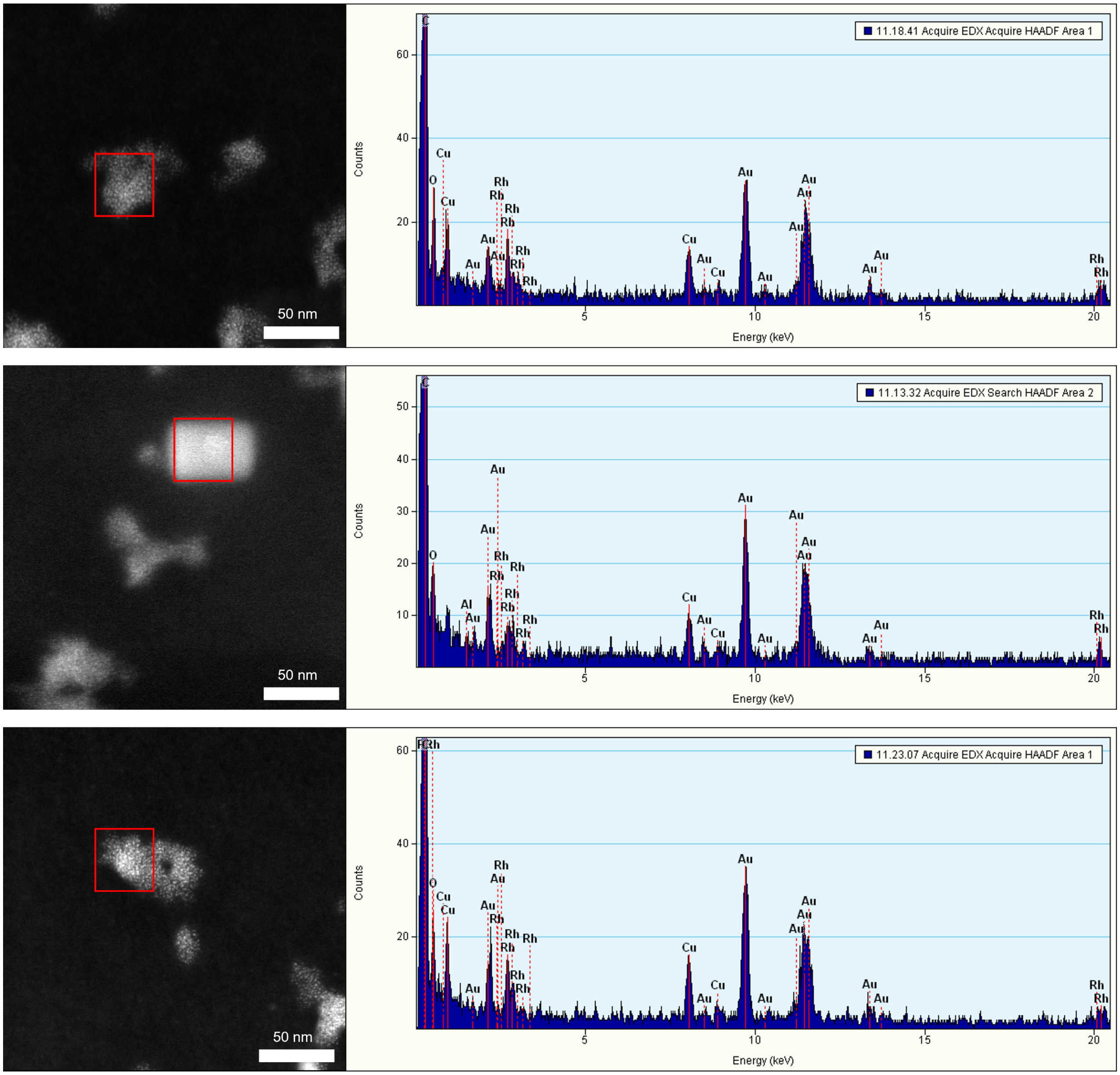

Figure S21. Representative HAADF-STEM images (left) and the corresponding EDX spectra (right) obtained from single RhCu-fdc-HKUST-1 crystalline particles. Note that Au signals stem from the TEM grid used for the measurements.

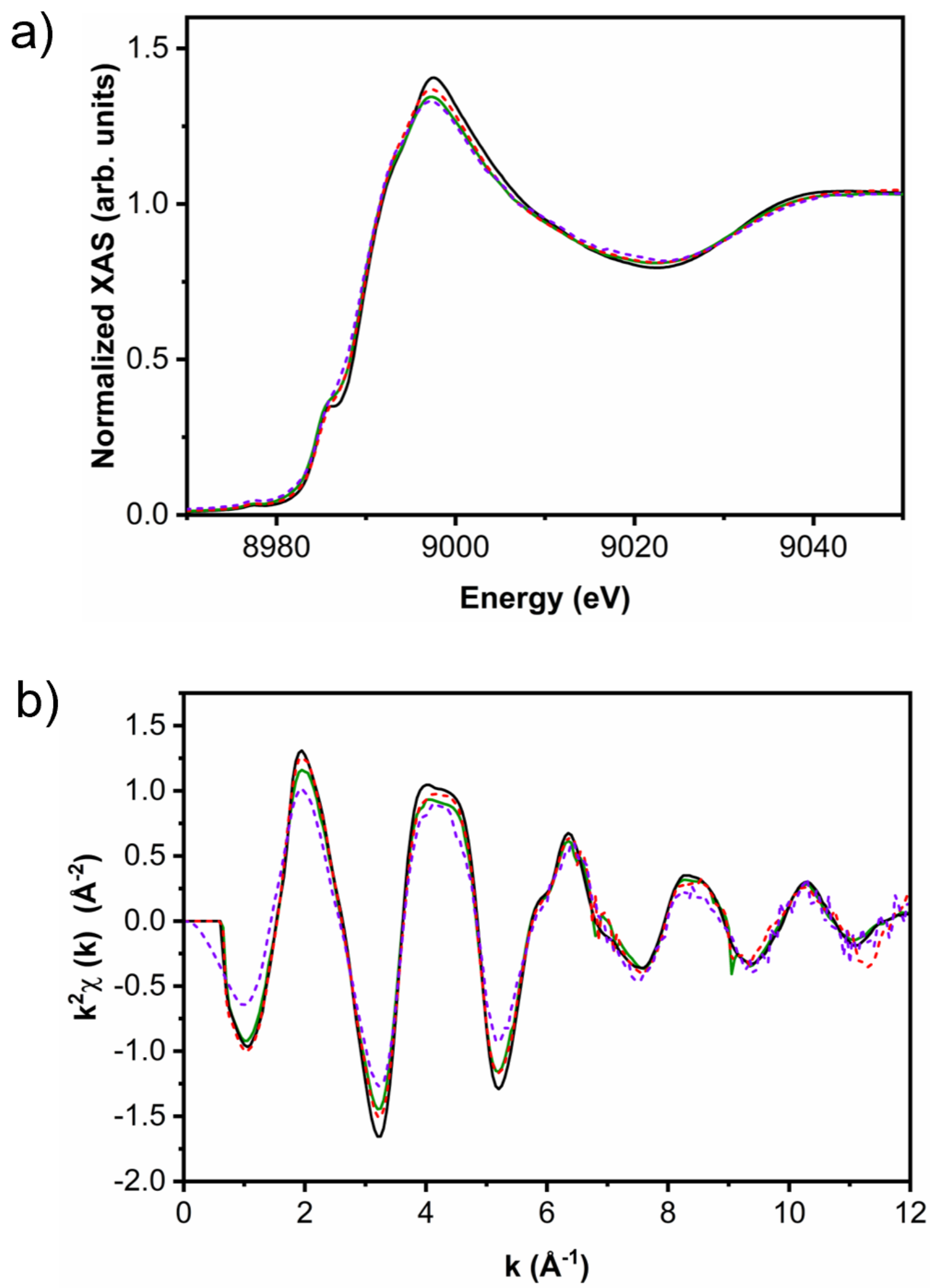

Figure S22. XAS analysis. (a) XANES spectra of RhCu–fdc–HKUST-1 (violet dashed line) compared to RhCu–bdc–HKUST-1 (dashed orange line), RhCu–btc–HKUST-1 (green line) and HKUST-1 (black line). (b) EXAFS spectra of RhCu–fdc–HKUST-1 (violet dashed line) compared to RhCu–bdc–HKUST-1 (dashed orange line), RhCu–btc–HKUST-1 (green line) and HKUST-1 (black line). These results confirm that the coordination environment of Cu(II) in RhCu-bdc-HKUST-1 and RhCu–fdc–HKUST-1 is consistent with that in HKUST-1.

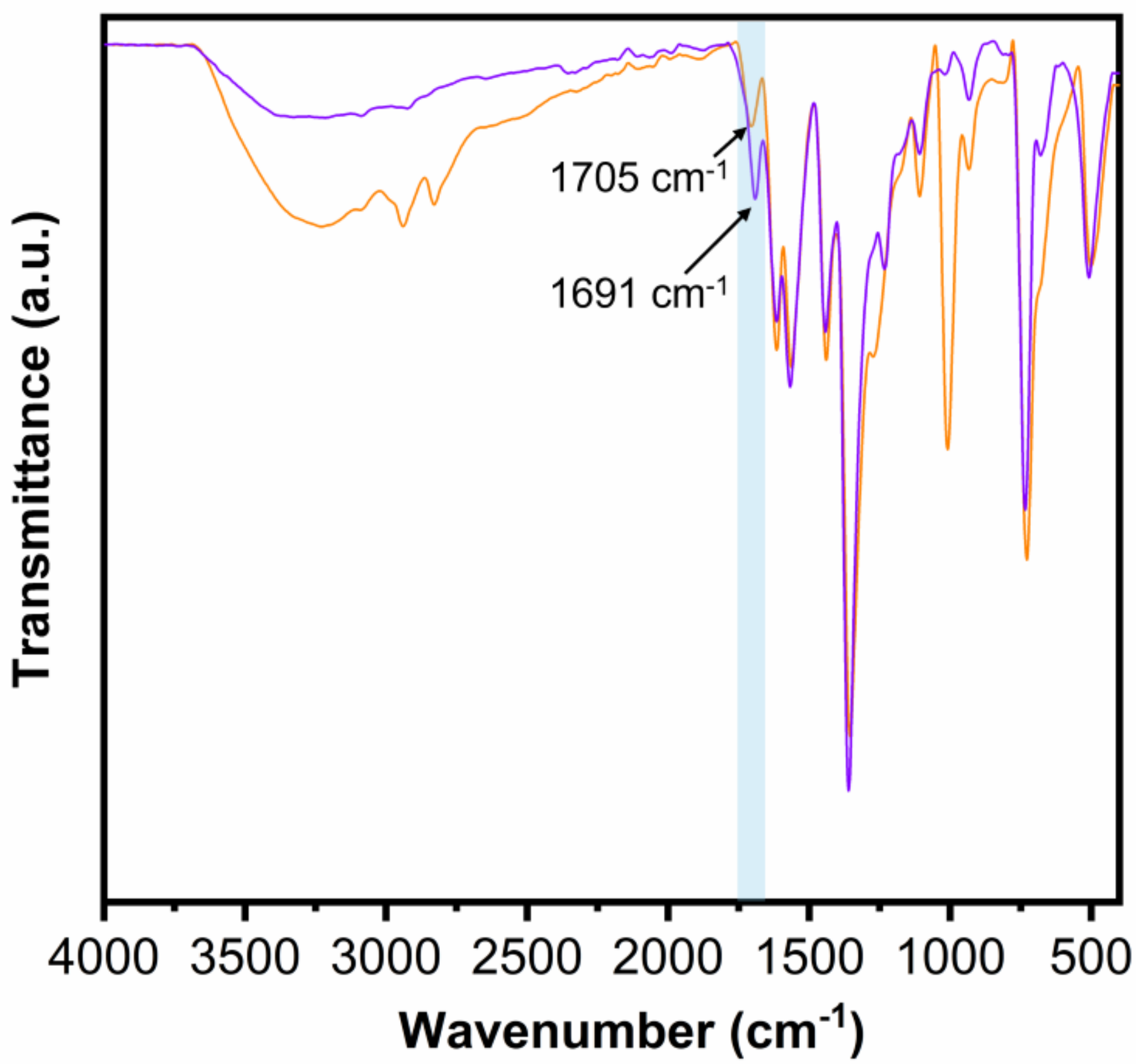

Figure S23. FTIR-ATR spectra of RhCu-bdc-HKUST-1 (orange line) and RhCu-fdc-HKUST-1 (violet line).

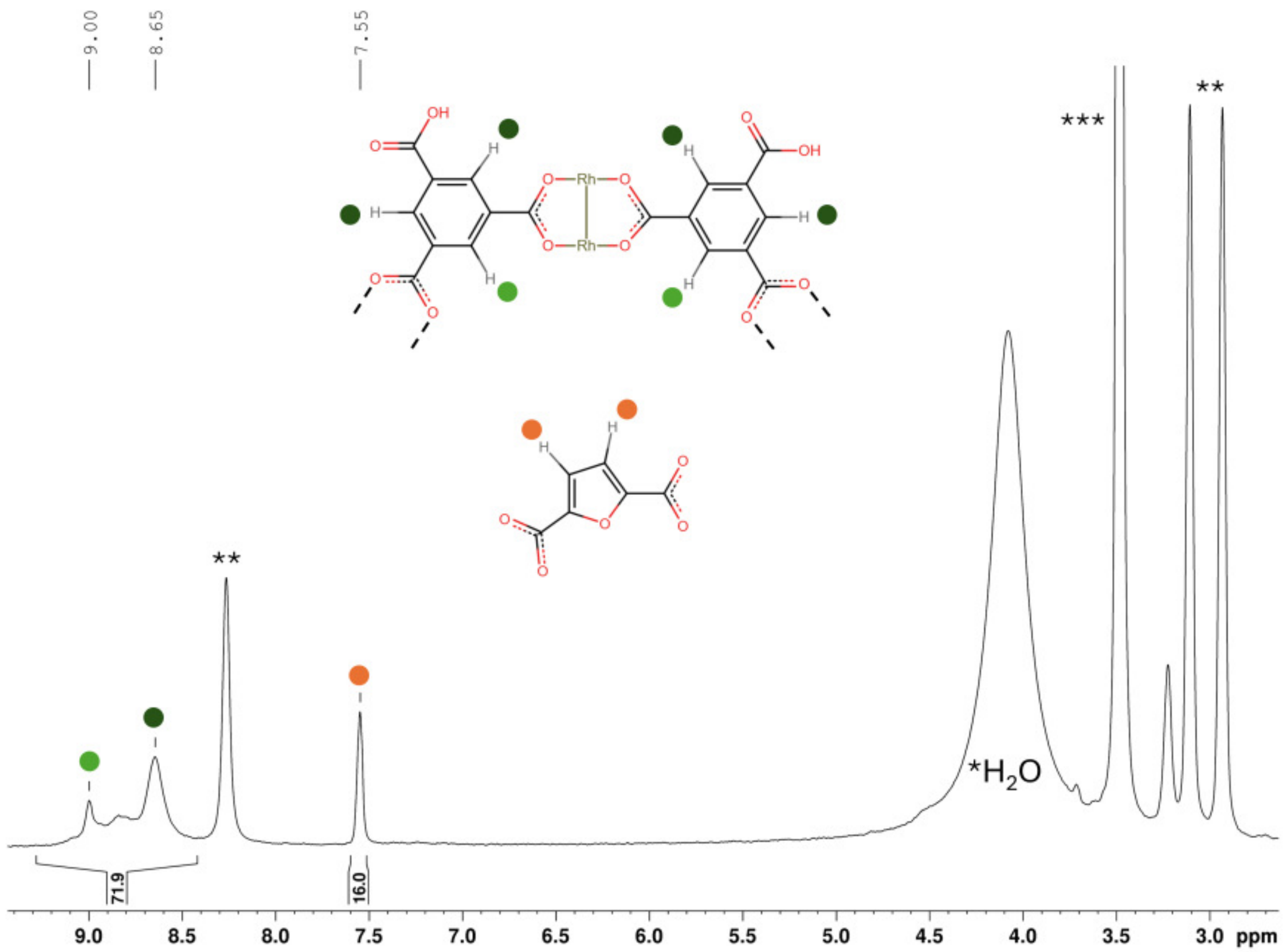

Figure S24. ^{1}H NMR spectrum (400 MHz, dmf-d7) of digested RhCu-fdc-HKUST-1, obtained after adding a DCl solution under stirring. ** indicates DMF and *** indicates MeOH. Note that in the proposed structure of RhCu-fdc-HKUST-1 there are 8 fdc ligands per COOH-RhMOP and 72 btc protons in each COOH-RhMOP. The observed integration ratios are in agreement with the expected formula.

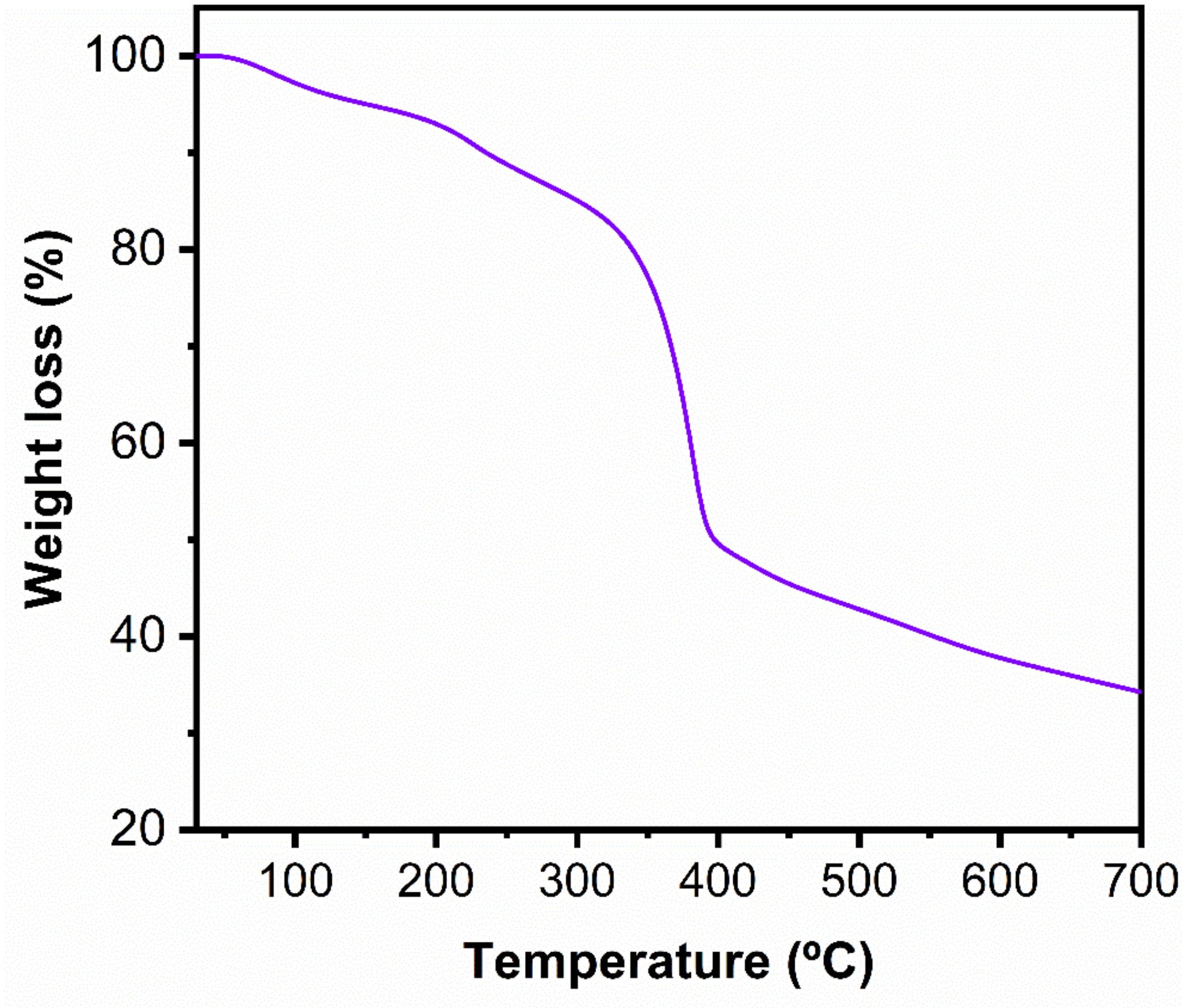

Figure S25. TGA of RhCu-fdc-HKUST-1. Note that the weight loss up to 200 ºC is associated to the remaining solvent molecules coordinated to the metal sites. The decomposition temperature of RhCu-bdc-HKUST-1 (340 ºC) is in good agreement to reported values for the parent HKUST-1 and RhCu-btc-HKUST-1.[5,6]

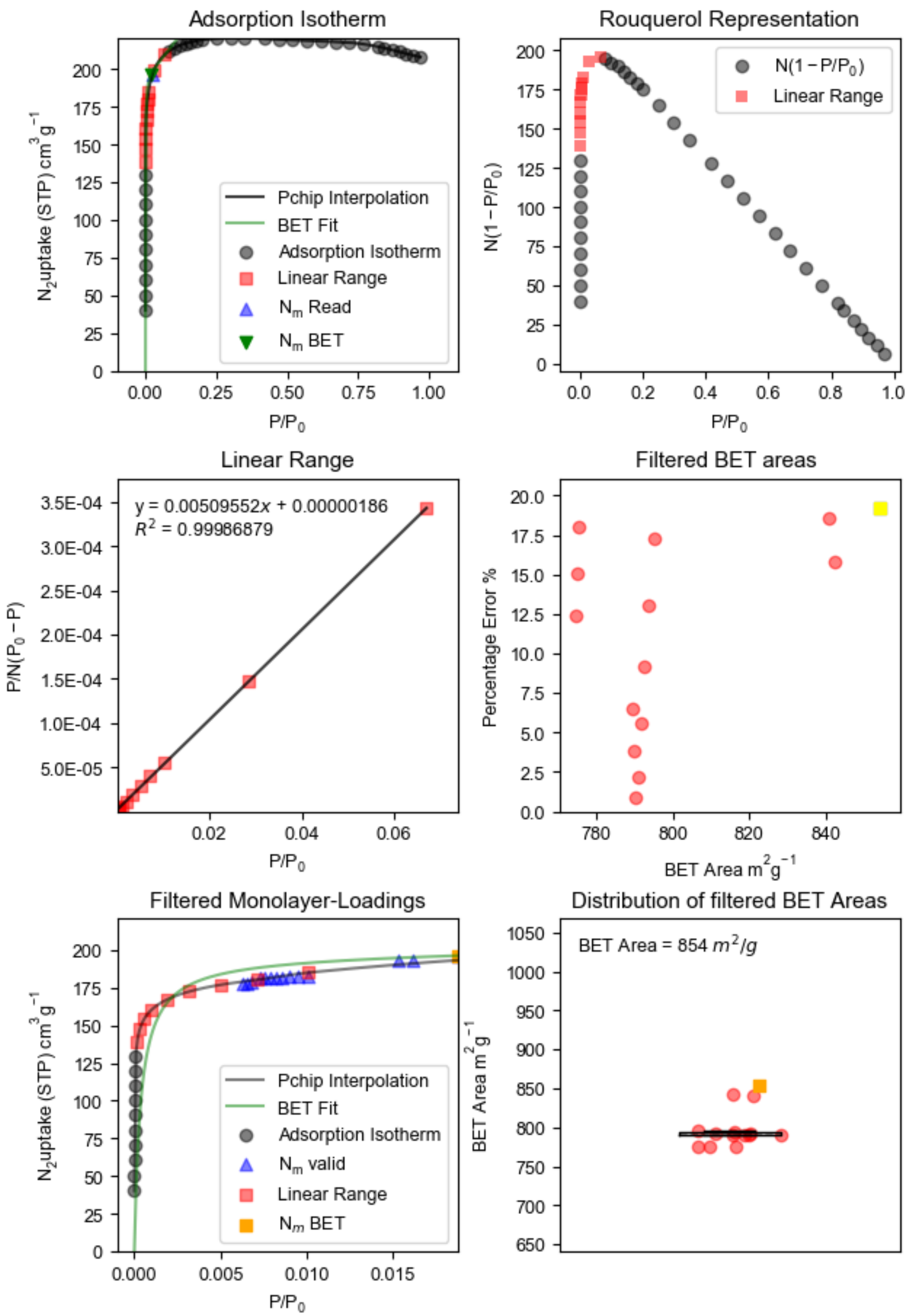

Figure S26. BETSI analysis of RhCu-bdc-HKUST-1.

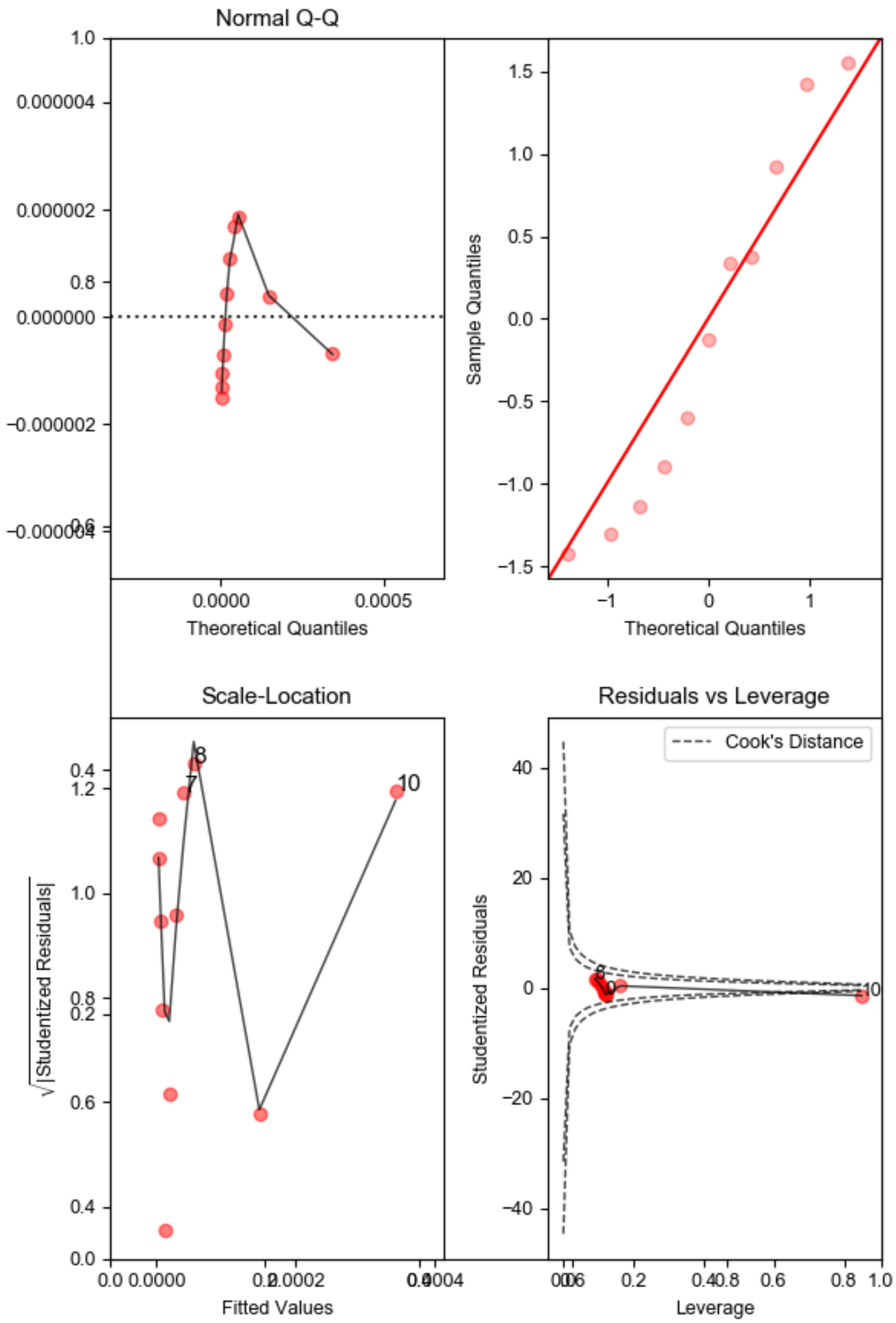

Figure S27. BETSI regression diagnostics for RhCu-bdc-HKUST-1.

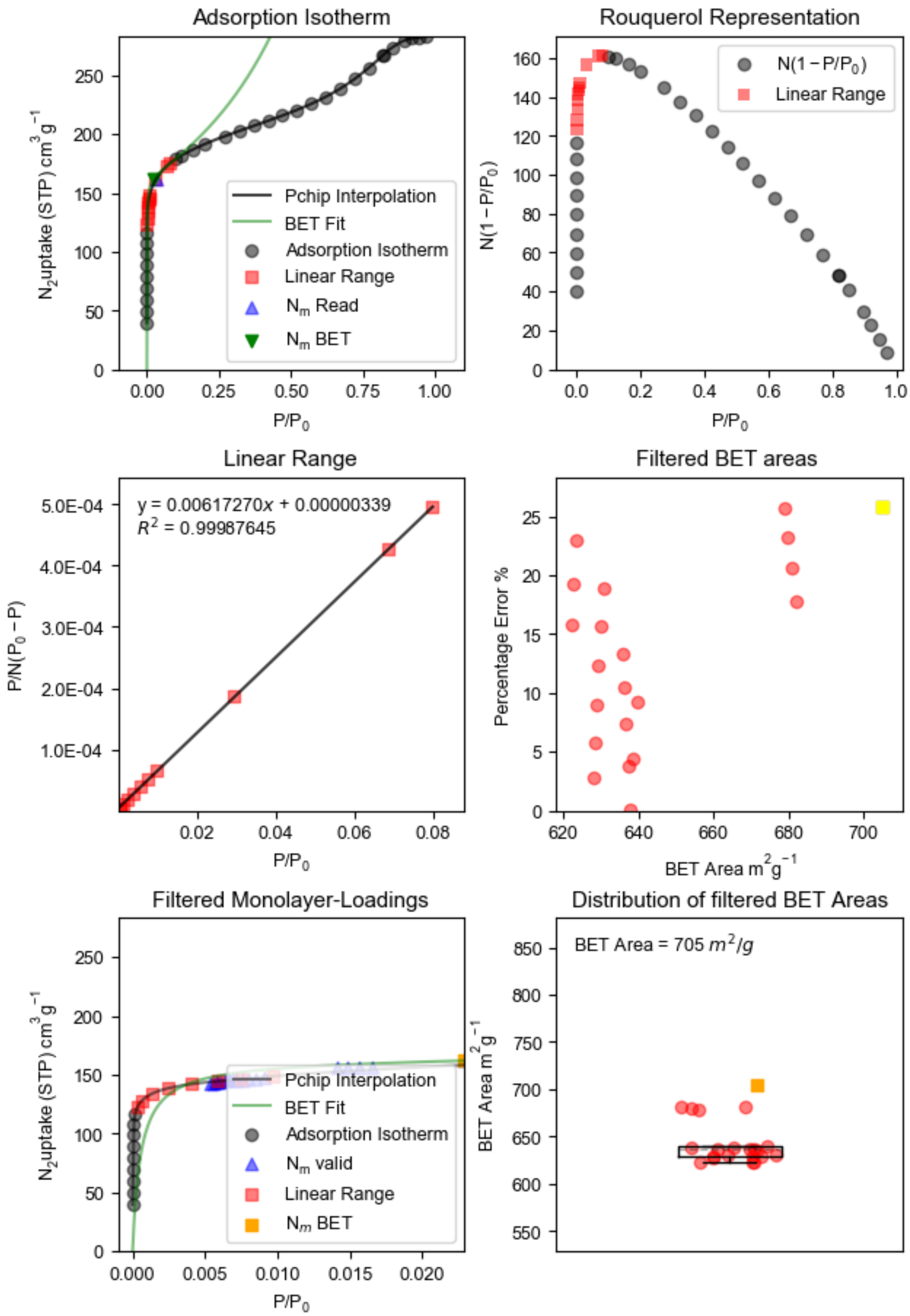

Figure S28. BETSI analysis of RhCu-fdc-HKUST-1.

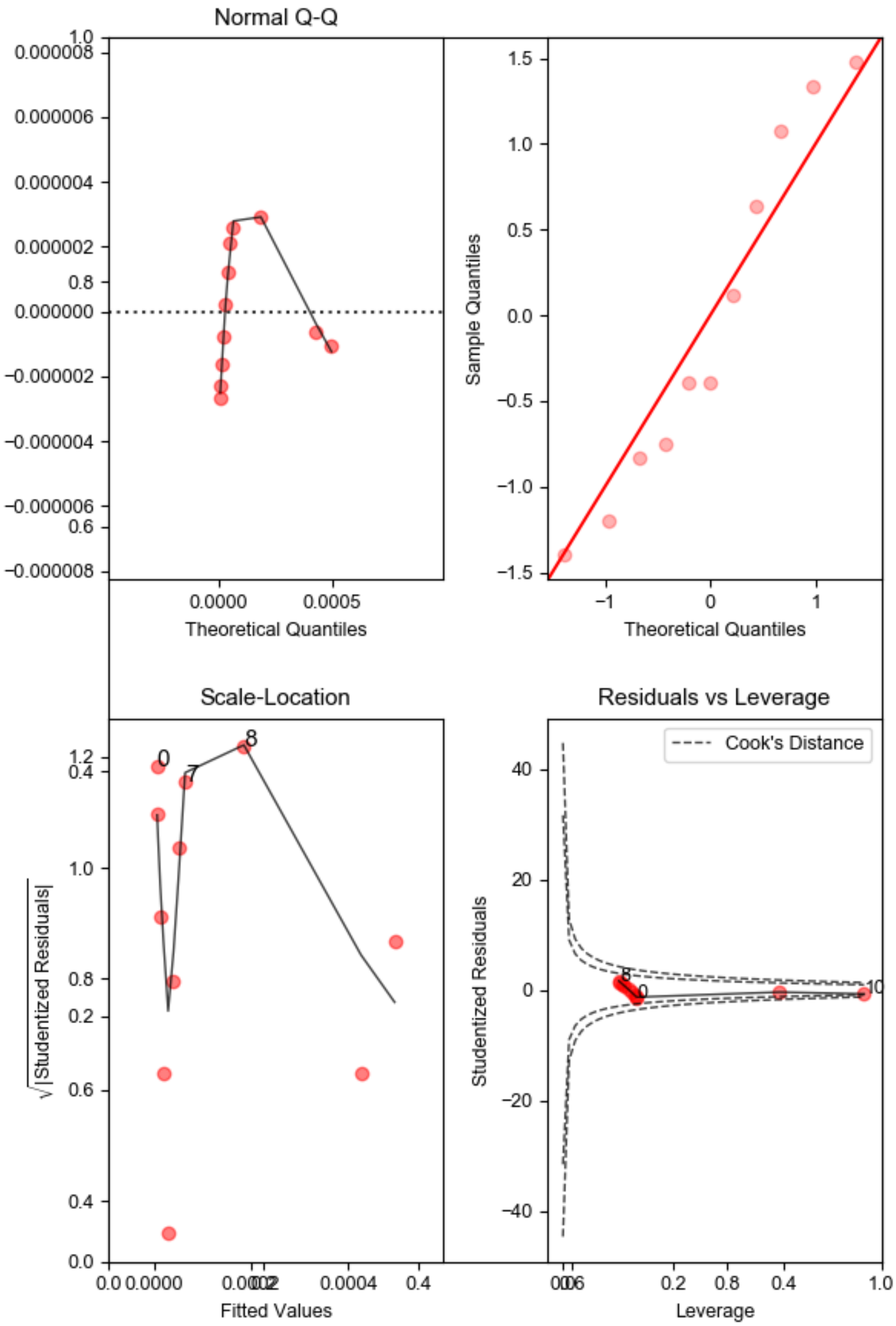

Figure S29. BETSI regression diagnostics for RhCu-fdc-HKUST-1.

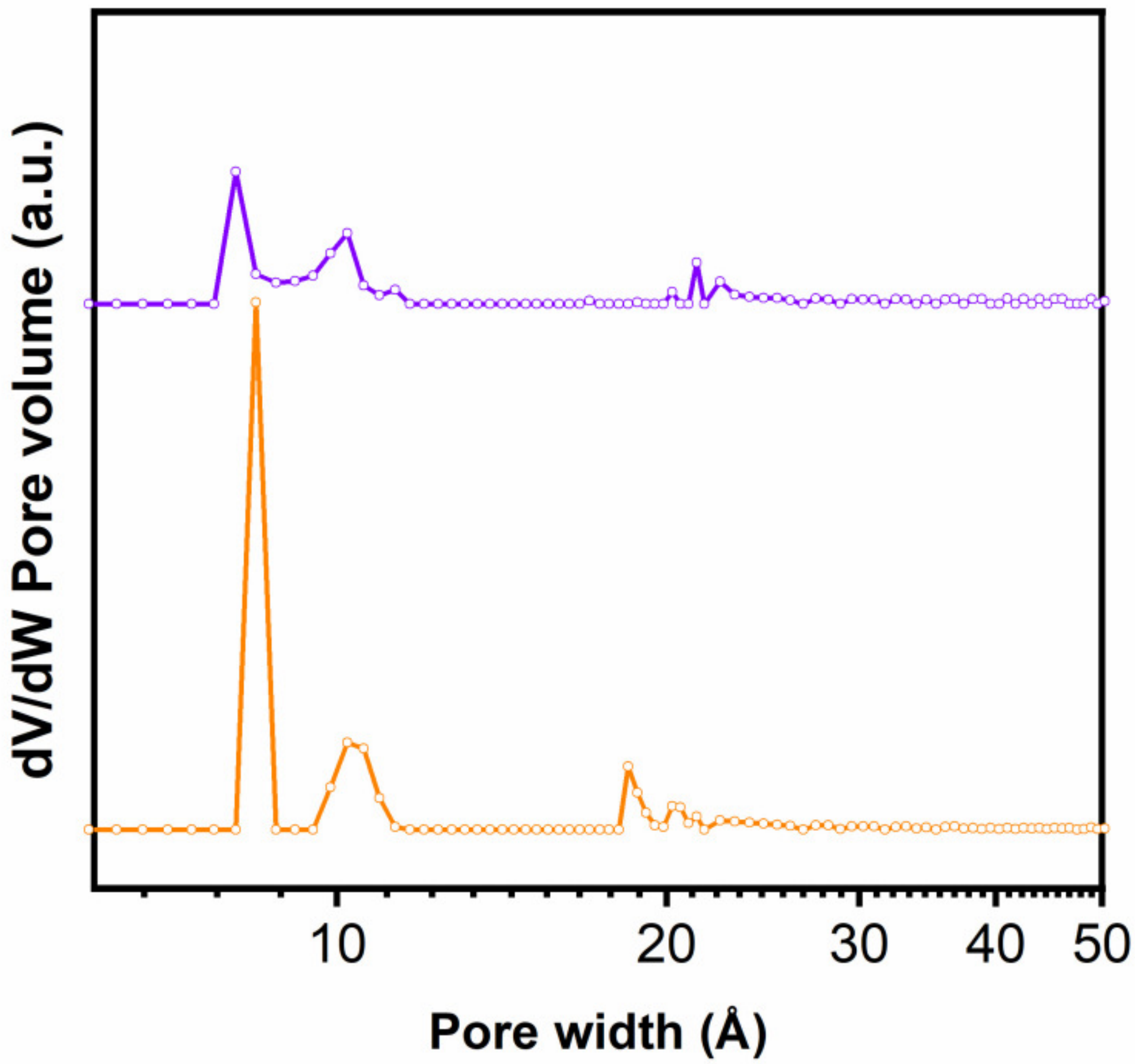

Figure S30. Pore size distribution of RhCu-bdc-HKUST-1 (orange) and RhCu-fdc-HKUST-1 (violet). Two main pore populations are observed at ~8 and ~10-11 Å, which are typically ascribed to the cuboctahedral cavities and to the octahedral cavities created by interconnected cuboctahedra. Additional pore populations in the 18–25 Å range confirm the presence of mesopores.

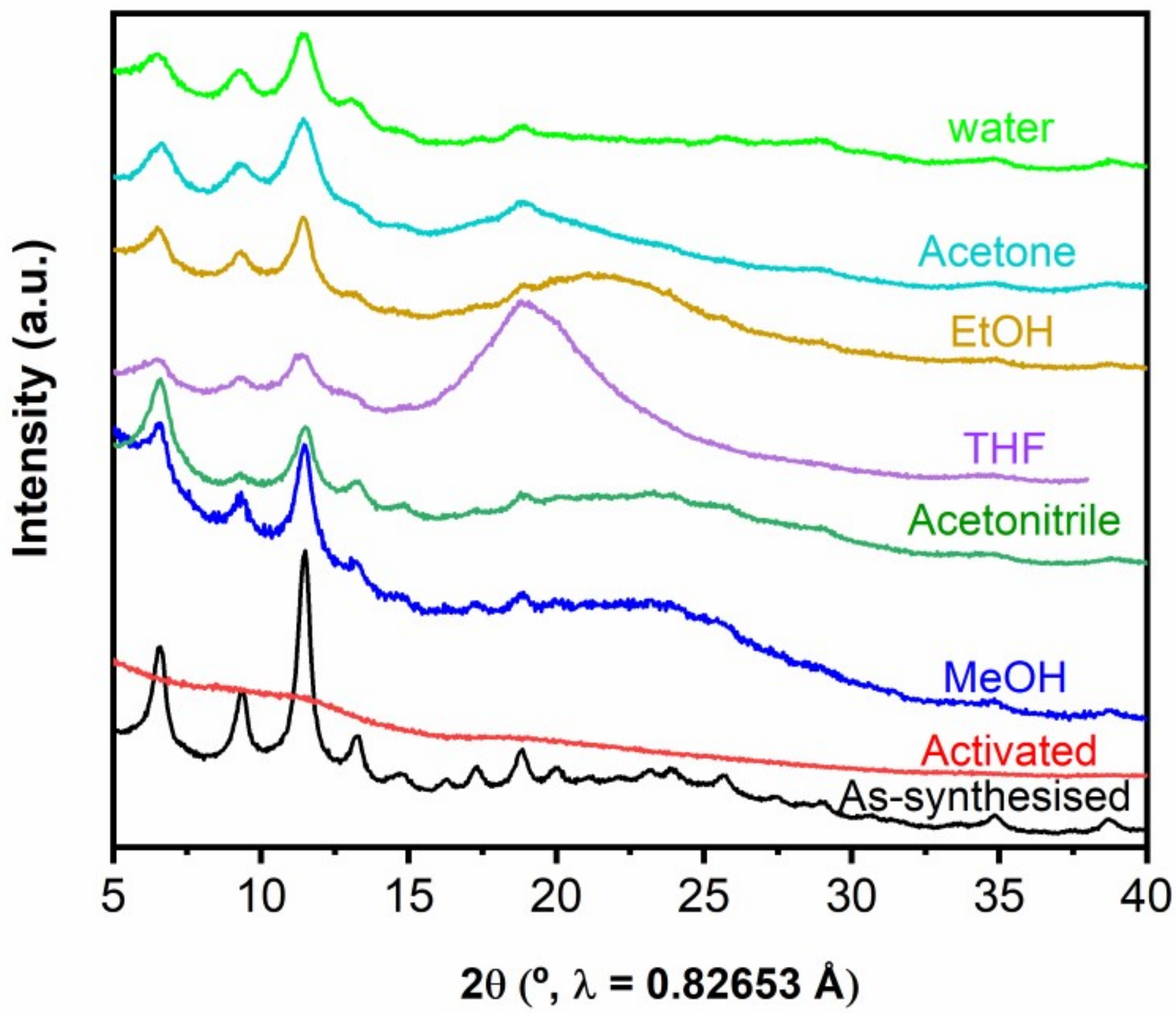

Figure S31. PXRD patterns of as-synthesised RhCu-bdc-HKUST-1 (black), after activation (red), and after being immersed in MeOH (blue), acetonitrile (green), THF (violet), EtOH (orange), acetone (turquoise), and water (light green).

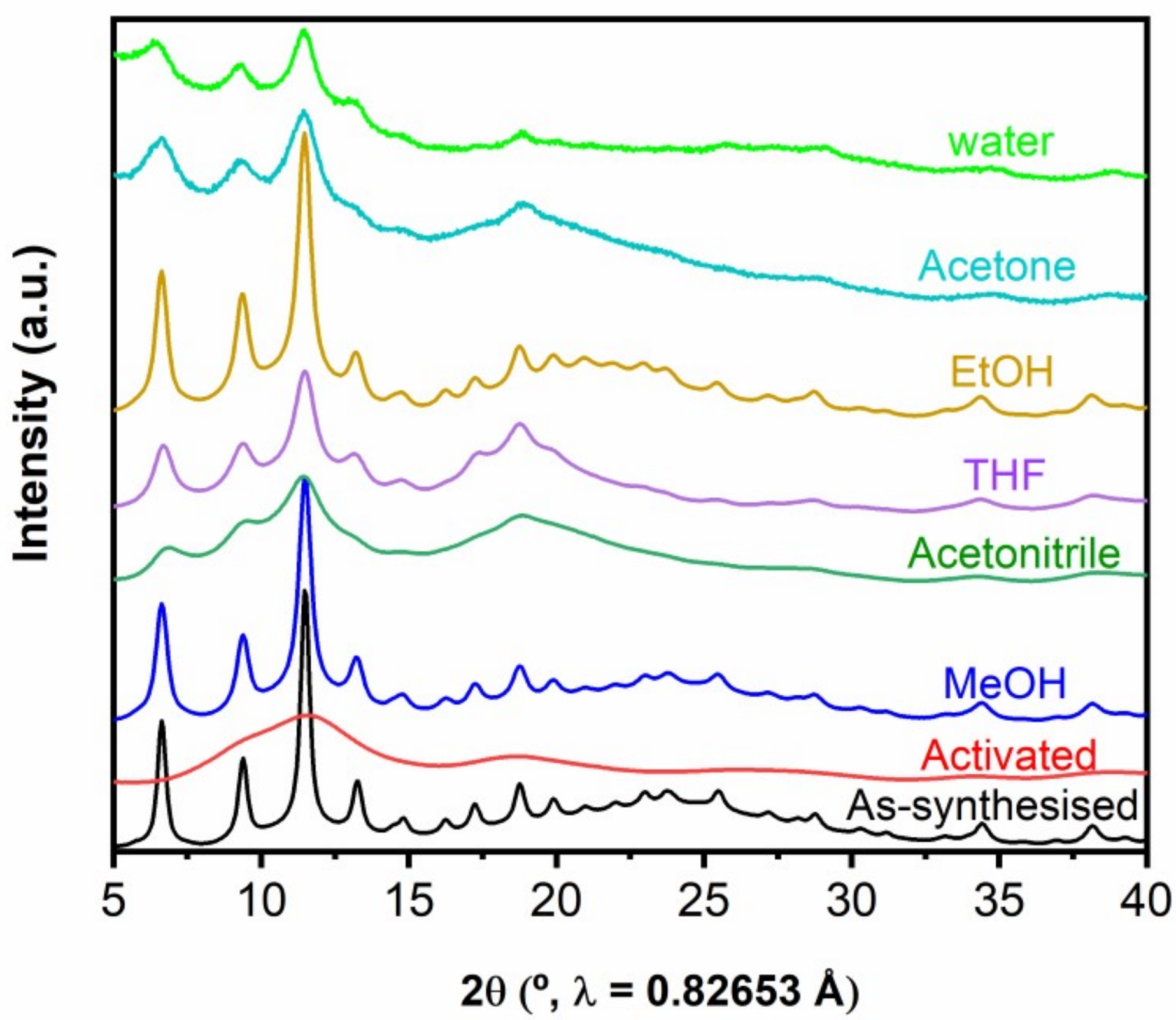

Figure S32. PXRD patterns of as-synthesised RhCu-fdc-HKUST-1 (black), after activation (red), and after being immersed in MeOH (blue), acetonitrile (green), THF (violet), EtOH (orange), acetone (turquoise), and water (light green).

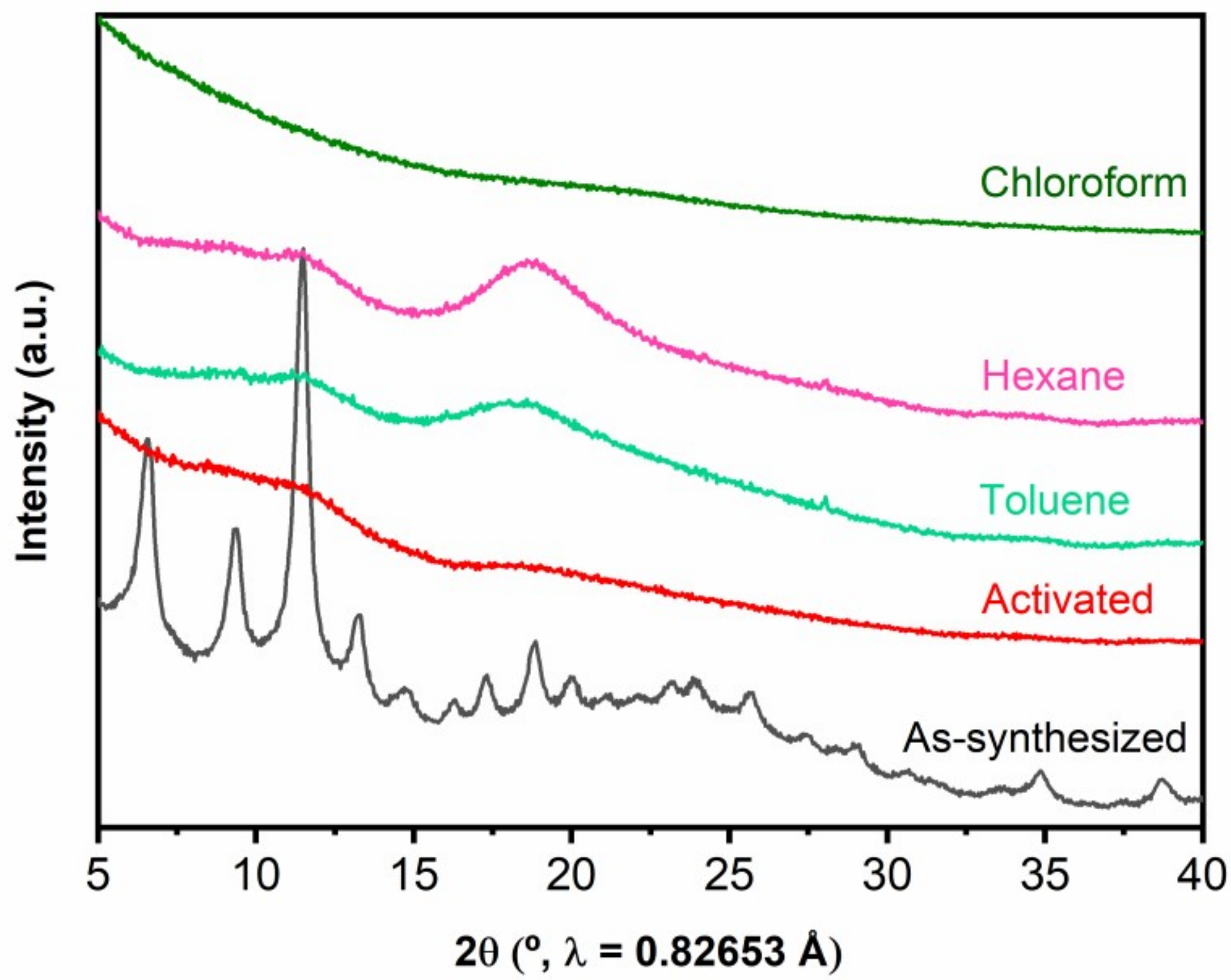

Figure S33. PXRD patterns of as-synthesised RhCu-bdc-HKUST-1 (black), after activation (red), and after being immersed in toluene (light green), hexane (pink) and chloroform (dark green).

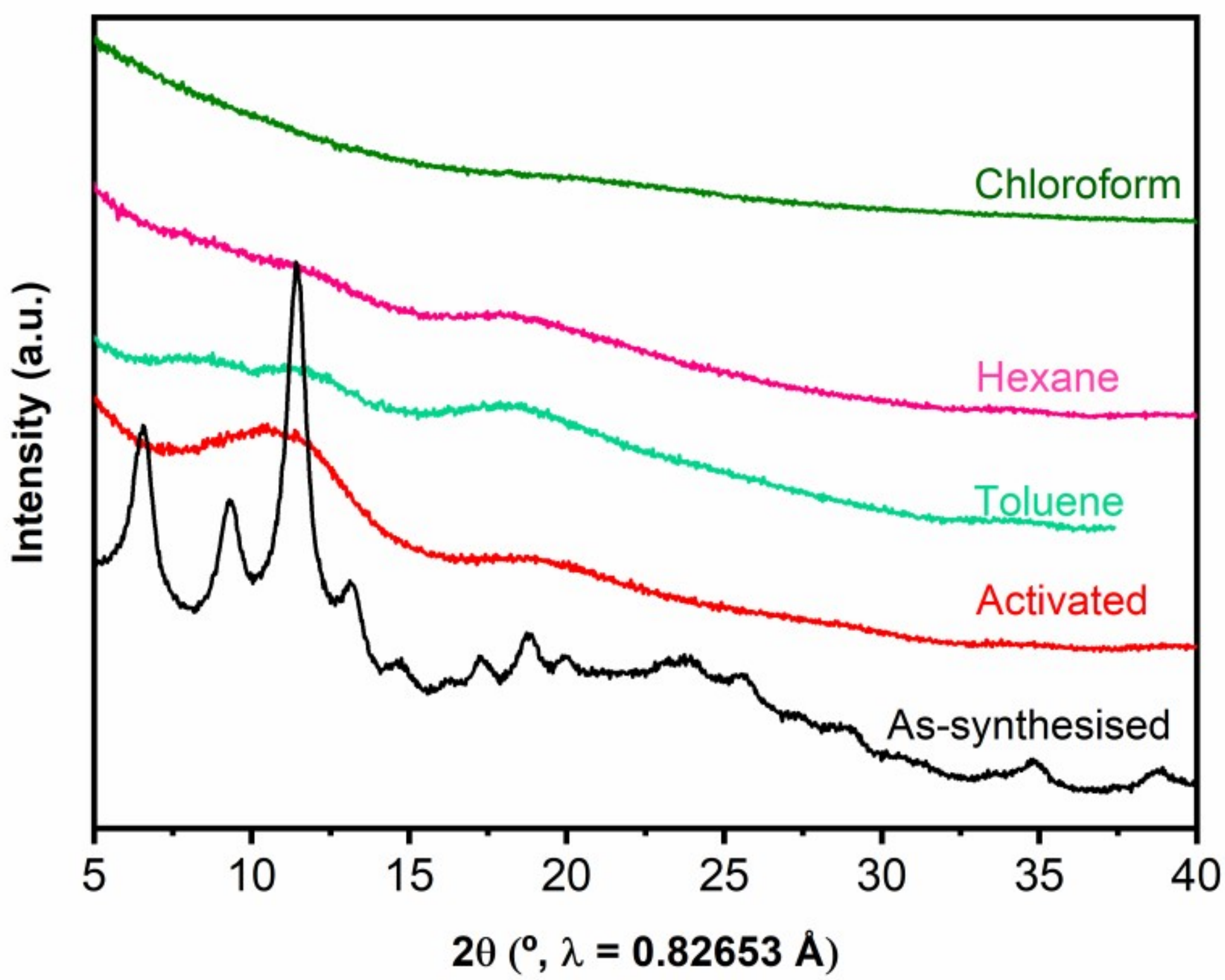

Figure S34. PXRD patterns of as-synthesised RhCu-fdc-HKUST-1 (black), after activation (red), and after being immersed in toluene (light green), hexane (pink) and chloroform (dark green).

References

[1] J. Juanhuix, F. Gil-Ortiz, G. Cuní, C. Colldelram, J. Nicolás, J. Lidón, E. Boter, C. Ruget, S. Ferrer, J. Benach, *J. Synchrotron Rad.* **2014**, *21*, 679-689

[2] A. P. Hammersley, *J. Appl. Cryst.* **2016**, *49*, 646-652

[3] J. Osterrieth, J. Rampersad, D. G. Madden, N. Rampal, L. Skoric, B. Connolly, M. Allendorf, V. Stavila, J. Snider, R. Ameloot, J. Marreiros, C. O. Ania, D. C. S. Azevedo, E. Vilarrasa-García, B. F. Santos, X.-H. Bu,; X. Zang, H. Bunzen, N. Champness, S. L. Griffin, B. Chen, R.-B. Lin, B. Coasne, S. M. Cohen, J. C. Moreton, Y. J. Colon, L. Chen, R. Clowes, F.-X. Coudert, Y. Cui, B. Hou, D. D'Alessandro, P. W. Doheny, M. Dinca, C. Sun, C. Doonan, M. Huxley, J. D. Evans, P. Falcaro, R. Riccò, O. K. Farha, K. B. Idrees, T. Islamoglu, P. Feng, H. Yang, R. Forgan, D. Bara, S. Furukawa, E. Sanchez, J. Gascon, S. Telalovic, S. K. Ghosha, S. Mukherjee, M. R. Hill, M. M. Sadiq, P. Horcajada, P. Salcedo-Abraira, K. Kaneko, R. Kukobat, J. Kenvin, S. Keskin, S. Kitagawa, K. Otake, R. P. Lively, S. J. A. DeWitt, P. L. Llewellyn, B. Lotsch, S. T. Emmerling, A. Pütz, C. Martí-Gastaldo, N. Muñoz, J. Garcia-Martinez, N. Linares, D. Maspoch, J. A. Suarez, P. Moghadam, R. Oktavian, R. Morris, P. Wheatley, J. Navarro, C. Petit, D. Danaci, M. Rosseinsky, A. Katsoulidis, M. Schroder, X. Han, S. Yang, C. Serre, G. Mouchaham, D. Sholl, R. Thyagarajan, D. Siderius, R. Q. Snurr, R. B. Goncalves, S. G. Telfer, S. J. Lee, V. Ting, J. Rowlandson,T. Uemura, T. Iiyuka, M. Veen, D. van der; Rega, V. Vanspeybroeck, A. Lamaire, S. Rogge, K. Walton, L. W. Bingel, S. Wuttke, J. Andreo, O. Yaghi, B. Zhang, C. Yavuz, T. Nguyen, F. Zamora, C. Montoro, H.-C. Zhou, K. Angelo, D. Fairen-Jimenez, *Adv. Mater.* **2022**, 2201502.

[4] A. J. Dent, G. Cibin, S. Ramos, A. D. Smith, S. M. Scott, L. Varandas, M. R. Pearson, N. A. Krumpa, C. P. Jones, P. E. Robbins, *J. Phys. Conf. Ser.* **2009**, *190*, 012039.

[5] B. Ravel, M. Newville, *J. Synchrotron Radiat.* **2005**, *12*, 537-541.

[6] J. Albalad, A. Carné-Sánchez, T. Grancha, L. Hernández-López, D. Maspoch, *Chem. Commun.* **2019**, *55*, 12785–12788.

[7] A. Kirchon, G. S. Day, Y. Fang, S. Banerjee, O. K. Ozdemir, H.-C. Zhou, *iScience* **2018**, *5*, 30–37.

[8] A. Khobotov-Bakishev, C. von Baeckmann, B. Ortín-Rubio, L. Hernández-López, A. Cortés-Martínez, J. Martínez-Esaín, F. Gándara, J. Juanhuix, A. E. Platero-Prats, J. Faraudo, A. Carné-Sánchez, D. Maspoch, *J. Am. Chem. Soc.* **2022**, *144*, 15745–15753.
